# Synthesis and Cytotoxic Activity Evaluation of New Cu(I) Complexes of Bis(pyrazol-1-yl) Acetate Ligands Functionalized with an NMDA Receptor Antagonist

**DOI:** 10.3390/ijms21072616

**Published:** 2020-04-09

**Authors:** Maura Pellei, Luca Bagnarelli, Lorenzo Luciani, Fabio Del Bello, Gianfabio Giorgioni, Alessandro Piergentili, Wilma Quaglia, Michele De Franco, Valentina Gandin, Cristina Marzano, Carlo Santini

**Affiliations:** 1 School of Science and Technology, Chemistry Division, University of Camerino, via S. Agostino 1, 62032 Camerino (MC), Italy; maura.pellei@unicam.it (M.P.); luca.bagnarelli@unicam.it (L.B.); lorenzo.luciani@unicam.it (L.L.); carlo.santini@unicam.it (C.S.); 2 School of Pharmacy, Medicinal Chemistry Unit, University of Camerino, via S. Agostino 1, 62032 Camerino (MC), Italy; gianfabio.giorgioni@unicam.it (G.G.); alessandro.piergentili@unicam.it (A.P.); wilma.quaglia@unicam.it (W.Q.); 3 Department of Pharmaceutical and Pharmacological Sciences, University of Padova, via Marzolo 5, 35131 Padova, Italy; michele.defranco@unipd.it (M.D.F.); cristina.marzano@unipd.it (C.M.)

**Keywords:** copper complexes, antitumor activity, metal-based drugs, bifunctionalized ligands, NMDA receptor ligand

## Abstract

In the present article, copper(I) complexes of bis(pyrazol-1-yl) carboxylic acid (LH), bis(3,5-dimethylpyrazol-1-yl) carboxylic acid (L^2^H), and bis(pyrazol-1-yl) acetates conjugated with an *N*-methyl-d-aspartate (NMDA) receptor antagonist (L^NMDA^ or L^2NMDA^) and phosphane ligands (triphenylphosphine or 1,3,5-triaza-7-phosphaadamantane) were synthesized. The selection of an NMDA antagonist for the coupling with LH and L^2^H was suggested by the observation that NMDA receptors are expressed and play a role in different types of cancer models. All the new complexes showed a significant antitumor activity on a panel of human tumor cell lines of different histology, with cisplatin-sensitive, cisplatin-resistant, or multi-drug-resistant phenotype. Their half maximal inhibitory concentration (IC_50_) values were in the low- and sub-micromolar range and, in general, significantly lower than that of cisplatin. Interestingly, the fact that all the complexes proved to be significantly more active than cisplatin even in three-dimensional (3D) spheroids of H157 and BxPC3 cancer cells increased the relevance of the in vitro results. Finally, morphological analysis revealed that the most representative complex **8** induced a massive swelling of the endoplasmic reticulum (ER) membrane, which is a clear sign of ER stress.

## 1. Introduction

Copper complexes are emerging as metal-based drug candidates for the treatment of cancer, due to their wide structural variability, biologically accessible redox properties, and bioavailability [1,2,3,4]. Part of this interest stems from the assumption that endogenous metal ions are less toxic to normal cells than non-endogenous metals. Actually, mammalians developed a series of specific and strictly controlled mechanisms to govern copper homeostatic levels, in order to maintain free copper ion concentration in cells and fluids <10^−18^ M. Once intracellularly absorbed, the potential toxicity of copper is handled by a complex regulatory network that allows copper distribution and managing [5].

Copper compounds display broader spectra of activities and lower toxicity, thereby providing the possibility of circumventing the problems encountered by clinically approved platinum drugs [6,7,8]. Furthermore, there is increasing evidence that the mechanism of action of copper complexes is distinctly different from that of Pt-based compounds, such as cisplatin [9] and, therefore, they might represent efficacious alternatives to platinum-based drugs [3,10].

Recent screenings on the anticancer potential of copper complexes showed promising in vitro and in vivo results [11,12,13,14,15,16,17,18,19,20], and they are currently receiving significant attention. Actually, the raised need for copper by cancer tissues and the established role of copper as a limiting factor for multiple aspects of tumor progression, including growth, angiogenesis, and metastasis, laid down the basis for the development of copper complexes as anticancer agents [12,21,22,23].

In the search for copper-based anticancer agents [1], over the last few decades, our attention was focused on the design and synthesis of copper complexes of bis(azol-1-yl) carboxylate heteroscorpionate ligands [24,25,26] of general formula [HC(COOH)(az)_2_], with az = pyrazole or 1,2,4-triazole [27,28,29,30]. Bis(azol-1-yl) carboxylic acids are convenient starting materials for the preparation of conjugated heteroscorpionate systems, due to the κ^3^-NNO coordination behavior of bis(azol-1-yl) methanes and to the presence of a carboxylic function suitable for the coupling with bioactive molecules [31,32,33,34,35,36]. We recently reported the synthesis and characterization of glucosamine and 5-nitroimidazole bifunctional ligands and the related Cu(I) and Cu(II) complexes, as well as a brief investigation of their cytotoxic activity toward human tumor cell lines [37,38,39].

**Scheme 1:**
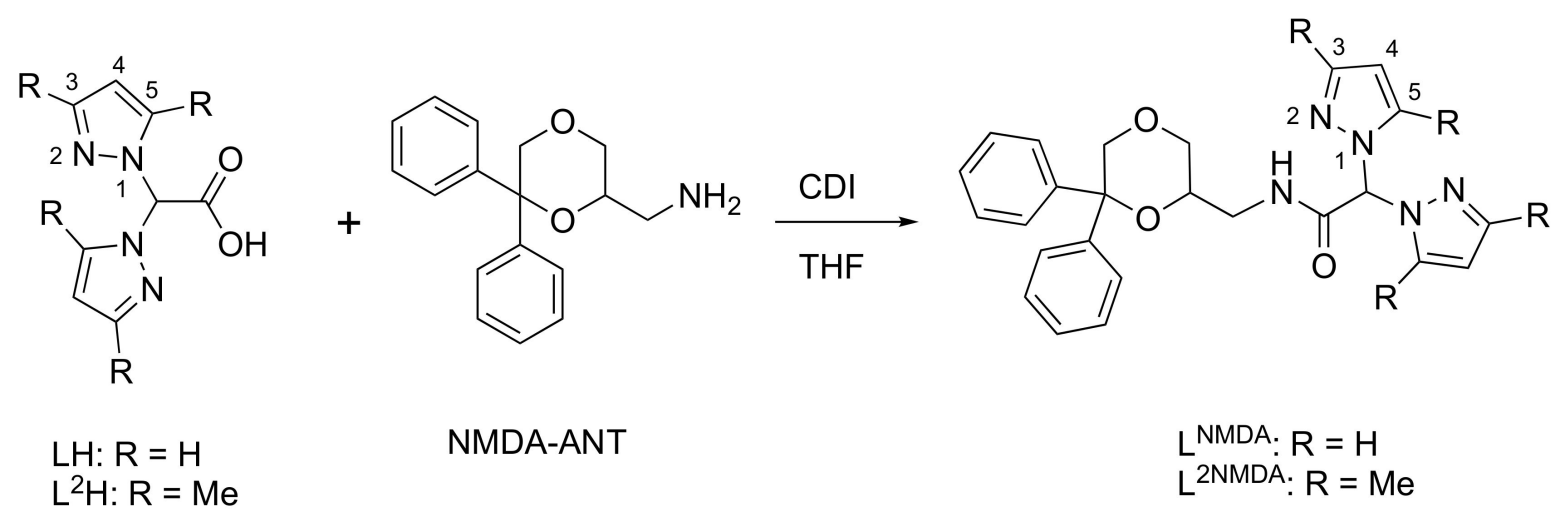
Reaction scheme for the synthesis of NMDA-ANT-conjugated bis(pyrazol-1-yl) acetate ligands L^NMDA^ and L^2NMDA.^

Among bioactive molecules, for the preparation of cytotoxic copper complexes, we also used an *N*-methyl-d-aspartate (NMDA) receptor ligand. NMDA receptors belongs to the family of ionotropic glutamate receptors and are cation channels with high calcium permeability, which are assembled by tetrameric combination of seven subunits, namely, GluN1, GluN2A-D, and GluN3A-B [40]. In addition to playing a critical role in the development of the central nervous system, they are also found in several types of cancer models, including colon, lung, and prostate cancer cell lines, as well as in gastric, esophageal, and hepatocellular carcinomas [41,42]. Moreover, it was demonstrated that breast cancer MCF7 cells express NMDA receptors, and the noncompetitive NMDA receptor antagonist MK-801 reduced cell viability on both cell lines. MK-801 also inhibited tumor growth of MCF7 tumor xenografts in nu/nu mice, suggesting the active role played by NMDA receptor in breast cancer survival and growth [43]. We recently reported that Cu(II) complexes of bifunctional heteroscorpionate ligands (L^NMDA^ and L^2NMDA^, Scheme 1), obtained by conjugating bis(pyrazolyl) carboxylic acid (LH) and bis(3,5-dimethylpyrazolyl) carboxylic acid (L^2^H) with the NMDA receptor antagonist 6,6-diphenyl-1,4-dioxan-2-yl) methanamine (named NMDA-ANT for briefness; Scheme 1) [44], showed antiproliferative activity against a panel of human tumor cell lines. A synergistic mechanism of action, due to the presence of the NMDA moiety and copper(II) in the same chemical entity, was hypothesized for these compounds [45].

**Figure 1:**
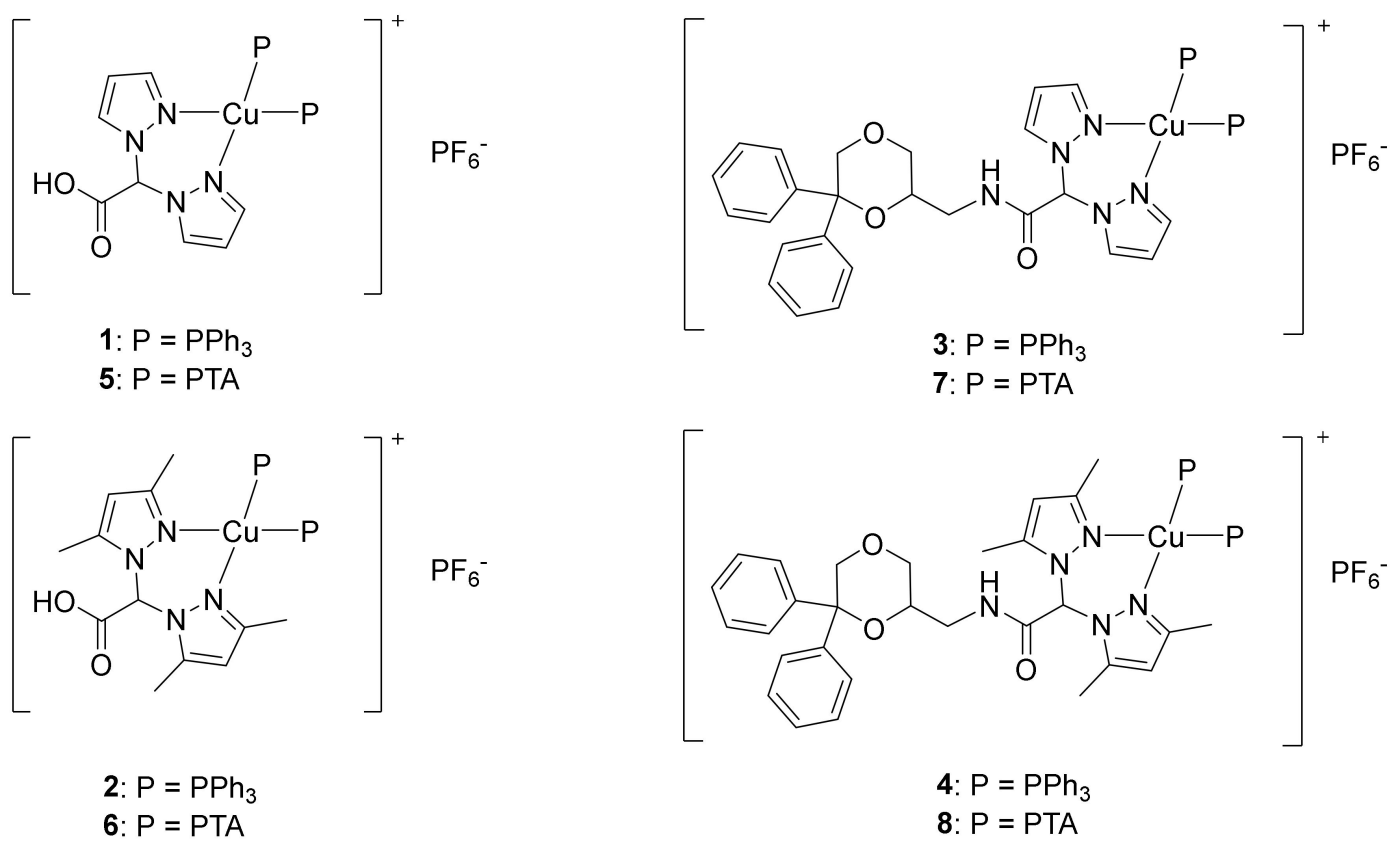
Chemical structures of complexes **1–8**.

Based on our experience [37] Cu(I) complexes might be more active anticancer agents than the corresponding Cu(II) ones [46,47]. Therefore, the aim of the present research was to synthesize and to assess the anticancer potential of novel Cu(I) complexes based on NMDA-ANT-conjugated bis(pyrazol-1-yl) acetate (L^NMDA^ or L^2NMDA^) and phosphane ligands (compounds **3**, **4**, **7**, and **8**; Figure 1), as well as the related Cu(I) phosphane complexes with the bis(pyrazol-1-yl) carboxylic acids LH and L^2^H (compounds **1**, **2**, **5**, and **6**; Figure 1). Phosphanes were used as coligands to stabilize copper in the +1 oxidation state. In particular, the lipophilic triphenylphosphine (PPh_3_) and the hydrophilic 1,3,5-triaza-7-phosphaadamantane (PTA) were selected to confer different solubility properties to the corresponding complexes.

The novel complexes **1–8** and the relative ligands were evaluated for their antitumor activity on a panel of human cancer cell lines, including cisplatin-sensitive and -resistant cells, as well as cells belonging to the multi-drug-resistant (MDR) phenotype. The inhibition of cancer cell viability was evaluated in both two-dimensional (2D) and three-dimensional (3D) experimental models. To investigate the correlation between the cytotoxic effect promoted by the complexes and their intracellular accumulation, cellular uptake experiments on the pancreatic BxPC3 cell line were performed. To preliminarily evaluate the potential molecular mechanism of the complexes, DNA interaction tests both in cell-free and in cellular systems were also performed. In addition, given that copper is a metal active from the redox point of view, the ability of the complexes to increase basal cell levels of reactive oxygen species (ROS) and, therefore, to alter cellular redox homeostasis was evaluated. Finally, the morphological and ultrastructural changes induced by the compounds were characterized by transmission electron microscopy (TEM).

## 2. Results and Discussion

### 2.1. Synthesis and Characterization

Ligands L^NMDA^ and L^2NMDA^ were prepared according to the procedure reported in Scheme 1. The acids LH and L^2^H [25,26] were activated with carbonyldiimidazole (CDI) and then treated with the amine NMDA-ANT. After separation and purification by column chromatography, L^NMDA^ and L^2NMDA^ were obtained in a reasonable yield and purity [45].

The copper(I) complexes [(LH)Cu(PPh_3_)_2_]PF_6_ (**1**), [(L^2^H) Cu(PPh_3_)_2_]PF_6_ (**2**) [(L^NMDA^)Cu(PPh_3_)_2_]PF_6_ (**3**), and [(L^2NMDA^) Cu(PPh_3_)_2_]PF_6_ (**4**) were prepared from the reaction of PPh_3_, Cu(CH_3_CN)_4_PF_6_, and the ligands LH, L^2^H, L^NMDA^, and L^2NMDA^, respectively (Figure 1), following a one-pot synthesis in CH_3_CN solvent. All the compounds are soluble in CH_3_OH, CH_3_CN, CHCl_3_, and dimethyl sulfoxide (DMSO); complex **2** is also soluble in acetone, while both **1** and **2** show low solubility in pure H_2_O and in aqueous buffered solution pH 7.4 (conditions comparable to cell culture medium); complex **4** is also soluble in EtOH, Et_2_O, tetrahydrofuran (THF), CH_2_Cl_2_, EtOAc, and acetone, while it is insoluble in pure H_2_O and hexane.

The infrared (IR) spectra carried out on the solid samples of complexes **1–4** showed all the expected bands for the heteroscorpionate ligands and PPh_3_ coligands. The strong absorptions due to the C=O stretching were detectable at 1752 and 1740 cm^−1^ for **1** and **2**, respectively, with no significant variations with respect to the same absorptions detectable in the spectra of the free ligands. The same absorptions due to C=O stretching were detectable at 1681 and 1674 cm^−1^ for **3** and **4**, respectively, being slightly shifted at lower frequencies with respect to the free ligands. Peaks in the range 1616–1519 cm^−1^ were attributable to the C=C/C=N stretching, lightly shifted to higher wavenumbers if compared to the related free ligand absorptions, probably due to the effect of the ligand–metal coordination. 

The ^1^H-NMR spectra of **1** and **2–4** were recorded in DMSO and CDCl_3_ solution, respectively. Complexes **1–3** showed a single set of resonances for the pyrazole rings, indicating that the pyrazoles were equivalents, with a slight shift to higher frequencies due to the coordination to the metal center. A significant shift to higher frequencies of the N–*H* signal at δ 8.17 ppm for compound **3** was probably due to a secondary interaction between the hydrogen and the metal center. The PPh_3_ coligands showed a characteristic series of peaks in the aromatic region, with an integration, with respect to the ligand peaks, which confirmed the 1:2 stoichiometric ratio between the ligand and the PPh_3_. The room temperature (r.t.) ^31^P{H}-NMR spectra of **1** and **2** gave singlets at δ −3.77 and −2.85 ppm, respectively, downfield shifted with respect to the signal exhibited by the uncoordinated PPh_3_ coligand (δ = −6.86 ppm in DMSO solution), along with the characteristic septet centered at about δ −144 ppm due to the PF_6_^−^ counterion. The ^31^P{H}-NMR spectra of **3** and **4**, recorded in CDCl_3_ solution, gave a broad singlet at δ 0.25 and −3.85 ppm, respectively, downfield shifted with respect to the signal exhibited by the uncoordinated PPh_3_ coligand (δ = −5.36 ppm in CDCl_3_ solution), along with the characteristic septet centered at about δ −144 ppm due to the PF_6_^−^.

The formation of the complexes **1–4** was also confirmed by the presence in the positive-ion electrospray ionization (ESI) MS spectra of the peaks attributable to the [(LH)Cu(PPh_3_)]^+^, [(L^2^H)Cu(PPh_3_)]^+^, [(L^NMDA^)Cu(PPh_3_)]^+^, and [(L^2NMDA^)Cu(PPh_3_)_2_]^+^ species. In the positive-ion spectra of **1**, **3**, and **4**, it was also possible to detect the major peak attributable to the [Cu(PPh_3_)_2_]^+^ species. In the negative ion spectra of complexes **1–4**, the [PF_6_]^−^ ion was observed as the major peak for all the complexes. The elemental analyses confirmed the stoichiometry of the products in the solid state.

The water-soluble phosphane PTA was used in the reaction with Cu(CH_3_CN)_4_PF_6_ and the ligands LH, L^2^H, L^NMDA^, and L^2NMDA^ to synthesize the Cu(I) complexes [(LH)Cu(PTA)_2_]PF_6_ (**5**), [(L^2^H)Cu(PTA)_2_]PF_6_ (**6**), [(L^NMDA^)Cu(PTA)_2_]PF_6_ (**7**), and [(L^2NMDA^)Cu(PTA)_2_]PF_6_ (**8**), respectively (Figure 1). All the compounds obtained were soluble in CH_3_CN and DMSO; compounds **5** and **6** were also soluble in pure H_2_O.

The IR spectra carried out on the solid samples of **5–8** showed all the expected bands for the heteroscorpionate ligands and the PTA coligands. The absorptions due to the C=O stretching were detectable at 1645 and 1640 cm^−1^ for **5** and **6**, respectively, shifted at lower frequency, due to a possible interaction between the carboxylic groups of the ligands and the nitrogen atoms of the PTA (hydrogen bonds). The same absorptions were detectable at 1696 and 1699 cm^−1^ for **7** and **8**, respectively, with no significant variations with respect to the related absorptions detectable in the spectra of the free ligands. The C=C/C=N stretching absorptions for complexes **5–8** were in a range similar to that observed for the free ligands.

The ^1^H-NMR spectra of **5** and **6** showed a single set of resonances for the pyrazole rings, indicating that the pyrazoles were equivalent and the slight shift of the resonances was due to the coordination to the metal center. The PTA coligands showed a characteristic series of peaks with an integration of the peaks, with respect to the ligand resonances, which confirmed the 1:2 stoichiometric ratio between the ligand and the PTA. The methylene protons of the PTA upper rim fell at 4.10 and 4.12 ppm as broad singlets in the spectra of **5** and **6**, respectively. The magnetically non-equivalent methylene protons of the PTA lower rim appeared as a multiplet in the range 4.52–4.67 ppm for **5**, and in the range 4.53–4.82 ppm for **6**. The C*H*COO protons of **5** and **6** appeared at 7.29 and at 6.44 ppm, respectively. The ^1^H-NMR spectra of **7** and **8**, recorded in CDCl_3_ solution, showed all the signals attributable to the ligands L^NMDA^ and L^2NMDA^ and the PTA coligands, with similar patterns and minor shifts observed upon complex formation. The assignment of all protons was performed via C/H COSY spectra of selected compounds recorded in DMSO solution at r.t. (see Supplementary Materials).

The ^31^P{H}-NMR spectrum of [(LH)Cu(PTA)_2_]PF_6_ (**5**), recorded in D_2_O solution, gave a broad singlet centered at δ −87.01 ppm, along with the characteristic septet centered at δ −145.02 ppm, due to the PF_6_^−^ group. The signal at δ −87.01 ppm was downfield shifted with respect to the signal exhibited by the uncoordinated PTA ligand. The ^31^P{H}-NMR spectrum of [(L^2^H)Cu(PTA)_2_]PF_6_ (**6**), recorded in D_2_O solution, gave a singlet at δ −85.87 ppm, downfield shifted compared to the signal of the free PTA in the D_2_O solution (δ = −97.07 ppm), along with the characteristic septet centered at a δ −144.03 ppm due to the PF_6_^−^ counterion. The ^31^P{H}-NMR spectra of **7** and **8**, recorded in CD_3_CN solution, gave broad signals centered at −91.63 and −89.0 ppm, respectively, at higher frequencies with respect to the value of the free PTA in the CD_3_CN solution (δ = −102.07 ppm), in accordance with the presence of two PTA coligands in the metal coordination core. The characteristic septet centered at −143.52 ppm was due to the presence of PF_6_^−^.

The formation of complexes **5–8** was confirmed by the presence in the positive-ion ESI-MS spectra of the major peaks attributable to the [(LH)Cu(PTA)]^+^, [(L^2^H)Cu(PTA)]^+^, [(L^NMDA^)Cu(PTA)]^+^, and [(L^2NMDA^)Cu(PTA)]^+^ species. In the negative ion spectra of complexes **5–8**, [PF_6_]^−^ was observed as the major peak for all the complexes. The elemental analyses confirmed the stoichiometry and the purity of the products.

### 2.2. Biological Studies

The newly developed copper(I) complexes, as well as the uncoordinated ligands, were tested for their cytotoxic activity by means of the 3-(4,5-dimethylthiazol-2-yl)-2,5-diphenyltetrazolium bromide (MTT) assay, as reported in Section 3. The in-house human cancer cell line panel contained examples of pancreatic (PSN-1 and BxPC3), breast (MCF-7), lung (H157), cervical (A431), and ovarian (A2780) cancers. Cisplatin was used as reference compound and was tested under the same experimental conditions. The cytotoxicity parameters, expressed in terms of half maximal inhibitory concentration (IC_50_) after 72 h of drug exposure, are reported in Table 1.

Uncoordinated ligands LH, L^2^H, and L^2^NMDA proved to be scarcely effective against all tested cancer cell lines. On the contrary, NMDA-ANT and L^NMDA^ ligands showed a cytotoxic activity in the micromolar range. However, their cytotoxicity was significantly lower compared to that of the relative complexes **3**, **4**, **7**, and **8**.

Unfunctionalized complexes and those functionalized with NMDA-ANT were, on average, similarly active, suggesting that the ability of the ligand to bind NMDA receptor had a marginal role in the cytotoxicity of this class of complexes, with respect to that of copper. Such a hypothesis was supported by the observation that the complexes embedding the NMDA receptor antagonist (**3**, **4**, **7**, and **8**) did not show particularly higher activity toward cell lines expressing NMDA receptors. Interestingly, all of them were more potent than cisplatin against all tested cancer cell lines. Among the examined cell lines, pancreatic BxPC3 resulted to be the most sensitive one, with all derivatives **1–8** showing IC_50_ values ≤0.34 µM. Complex 3 was the most active functionalized compound, with average IC_50_ values against all cancer cells roughly 80 times better than those elicited by cisplatin and, in particular, against pancreatic BxPC3 cancer cells, with values more than 300 times lower. It is also interesting to note that, on average, complexes with PPh_3_ (**1–4**) showed activities comparable to those with PTA (**5–8**).

The human tumor cell line panel also included two cell lines selected for their resistance to cisplatin (A431-Pt and A2780cis) or belonging to the MDR phenotype (A2780-ADR) (Table 1). By comparing the RF values (where RF is the resistance factor, defined as the ratio between IC_50_ values calculated for the resistant cells and those arising from the corresponding sensitive cell line), it was proven that all new compounds were able to overcome cisplatin resistance and were not potential substrates for the P-gP. 

To further evaluate the anticancer potential of the new copper(I) compounds, they were also screened against 3D spheroids of lung (H157) and pancreatic (BxPC3) cancer cells. Conventional 2D cell cultures do not properly represent the complexity of in vivo tumors, thus scarcely predicting the in vivo anticancer activity. In contrast, 3D cell cultures possess several features that more closely mimic the heterogeneity and complexity of in vivo tumors, being consequently potentially more predictive for in vivo results [48]. The cancer spheroids were treated with copper(I) complexes or cisplatin for 72 h, and cell viability was assessed by means of the acid phosphatase (APH) assay (Table 2). Interestingly, all the compounds showed a growth-inhibitory effect significantly higher than cisplatin in both cell lines. In particular, against BxPC3 cells, the IC_50_ values of compounds **1–8** proved to be from 10-fold (compound **5**) to even 84-fold (compound **8**) lower than that of cisplatin.

**Figure 2:**
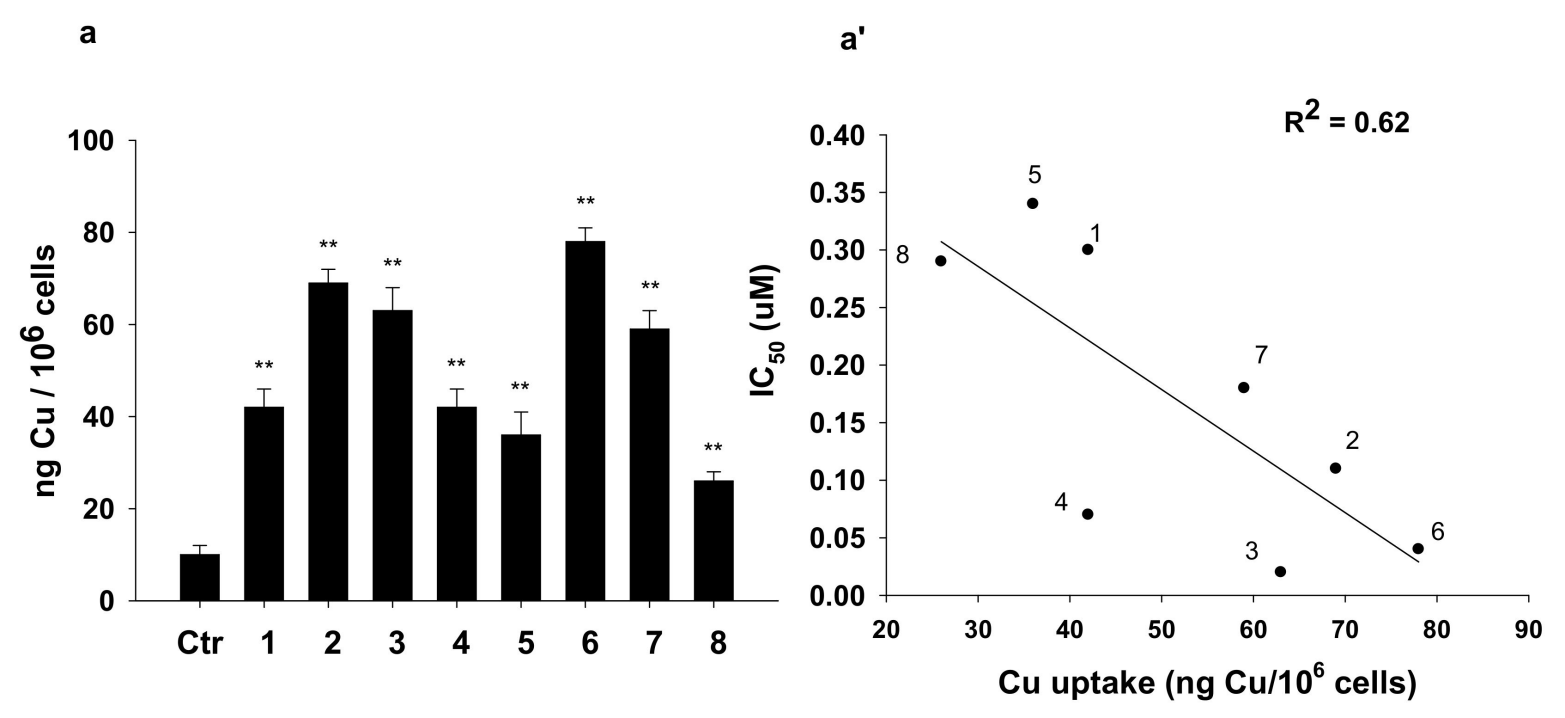
(**A**) Cellular uptake and (**B**) correlation between cytotoxicity and cellular copper levels in drug-treated BxPC3 cells. BxPC3 cells were incubated for 24 h with 1 μM of tested complexes. The amount of cellular Cu was estimated by Graphite Furnace Atomic Absorption Spectrometry (GF-AAS). The error bars indicate the SD; ** *p* < 0.01.

With the aim to correlate the cytotoxic potential of **1–8** with their ability to enter cancer cells, we evaluated cellular uptake in BxPC3 cancer cells treated for 24 h with equimolar concentrations (1 μM) of tested compounds (Figure 2). All compounds were able to cross cellular plasmalemma, and copper accumulated in treated cancer cells without a strict dependence on the lipophilic character of the whole Cu(I) complex. This could be in relation to the involvement of a carrier-mediated transport mechanism, as evidenced for other classes of cytotoxic Cu(I) species. A partially direct and linear correlation between the cytotoxic activity and the cellular uptake of the tested compounds was found (*R^2^* = 0.62).

As many studies identified DNA as the main molecular target for copper complexes [1], we firstly evaluated the ability of the newly developed Cu(I) complexes and related free ligands to interact with isolated circulating tumor DNA (ctDNA) via ultraviolet (UV) electronic absorption titrations. Upon increasing the concentration of compounds **1–8**, a dose-dependent modification of the DNA absorption bands was detected (see Supplementary Figure S1). Interestingly, free ligands were also slightly effective in inducing a modification of DNA spectra, thus indicating their ability to interact with DNA in cell-free conditions. In particular, a slight blueshift effect was evidenced for complexes with free or functionalized L^2^H ligand, notably **2**, **6**, **4**, and **8**, indicative of a partial grove binding and partial destabilization of the DNA chain.

**Figure 3:**
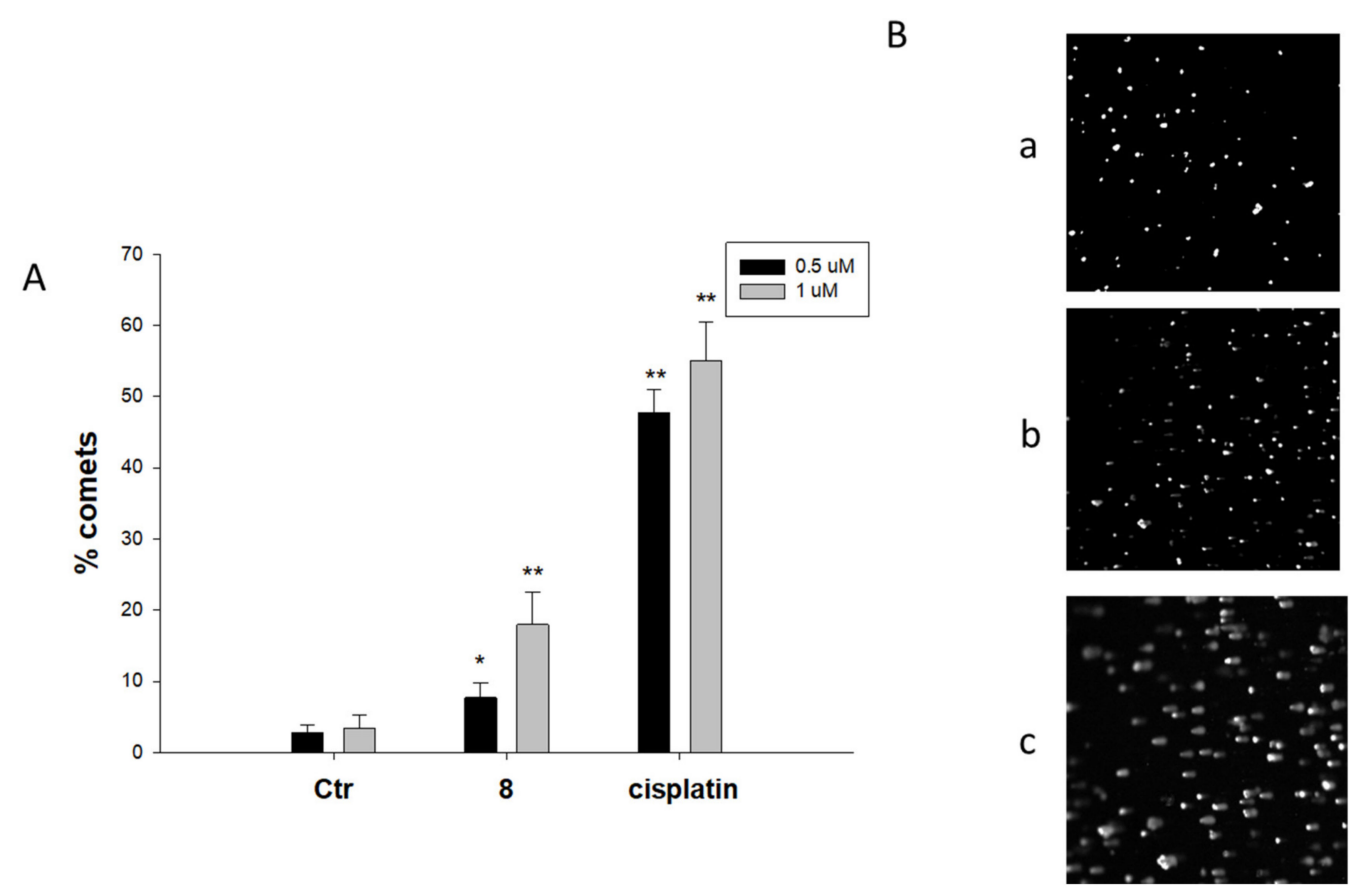
Comet assay. BxPC3 cells were treated for 3 h with 1 μM of compound **8** or cisplatin and then processed for comet assay. (**A**) Number of cells with well-formed comets measured by ImageJ software. The error bars indicate the SD; * *p* < 0.05, ** *p* < 0.01. (**B**) Representative images (100×) of control BxPC3 cells (a) or cells treated with 1 μM of **8** (b) and cisplatin (c).

On the other hand, cell studies performed using alkaline single-cell gel electrophoresis (Comet assay) revealed that complex **8**, the most active against 3D spheroids of BxPC3 cells, was only slightly effective in inducing cleavage of nuclear DNA in BxPC3 human pancreatic cancer cells, being significantly less effective than cisplatin even at the higher dose (Figure 3). In particular, cells treated with 1 μM of **8** displayed an increase of about 18% of well-formed comets compared with 55% comet formation induced by the reference metallodrug.

Copper complexes are regarded as redox active compounds and redox modulators. Actually, copper complexes may catalyze hydrogen peroxide in the form of Fenton-like reactions inside the cell to produce ROS, altering cellular redox homeostasis and, thus, driving cells toward oxidative stress [1]. On this basis, we evaluated the ability of complexes **1–8** to increase the cellular basal production of ROS in BxPC3 cancer cells.

**Figure 4:**
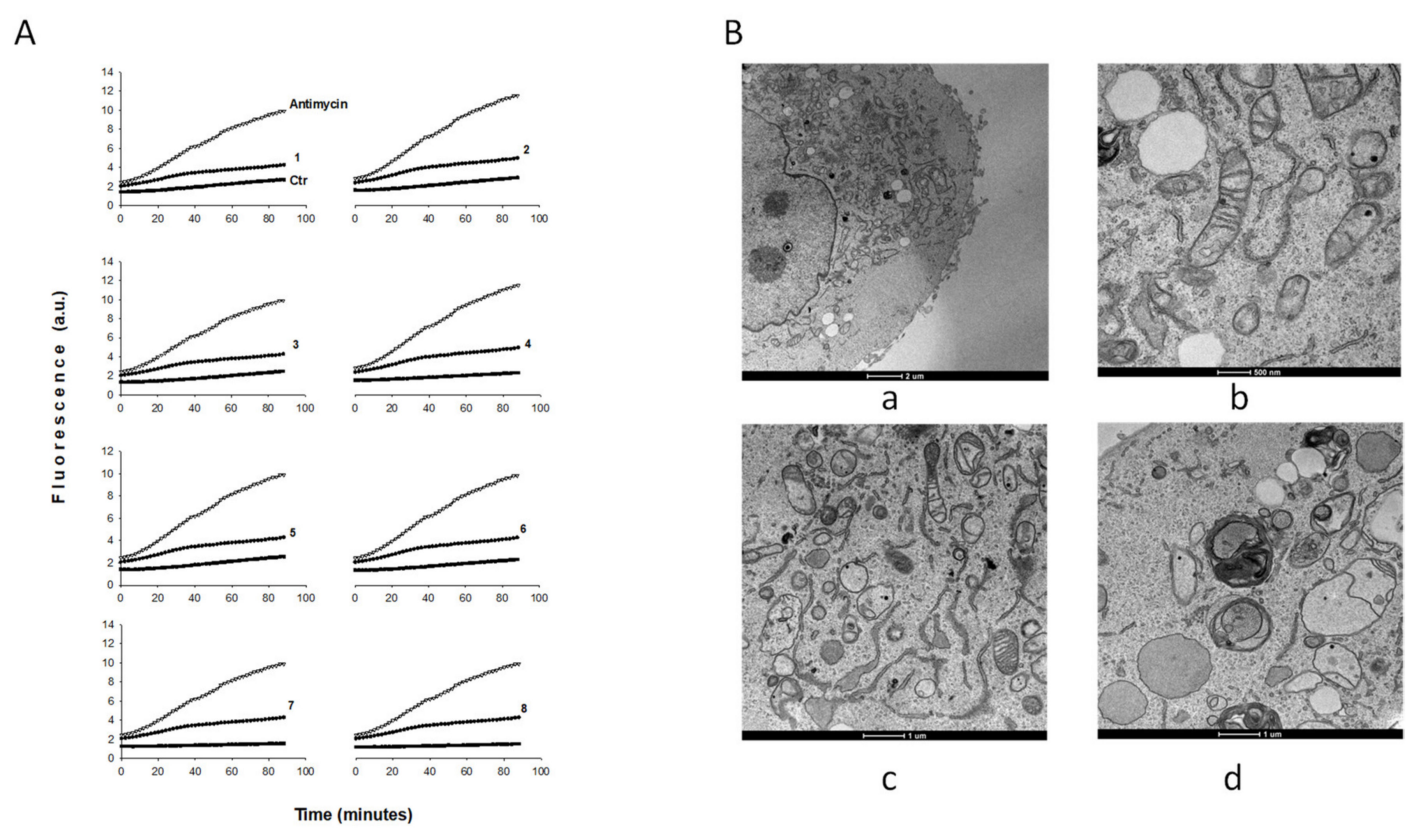
Reactive oxygen species (ROS) production and morphological changes. (**A**) Effect of compounds **1–8** on hydrogen peroxide formation in BxPC3 cells. BxPC3 cells were pre-incubated in phosphate-buffered saline (PBS)/10 mM glucose medium for 20 min at 37 °C in presence of 10 µM 5-(and-6)-chloromethyl-2′,7′-dichlorodihydrofluorescein diacetate acetyl ester (CM-DCFDA) and then treated with the IC_50_ of copper(I) compounds as a function of time (minutes). (**B**) TEM analysis. Transmission electron micrographs of BxPC3 cells after 24 h or 48 h of treatment with complex **8:** a and b, controls; c, 24 h compound **8**; d, 48 h compound **8**.

Notably, treatment with **1–8** determined a slight but not significant time-dependent increase in basal hydrogen peroxide formation (Figure 4, panel A), thus excluding the occurrence of significant oxidative stress. On the contrary, antimycin, a classic inhibitor of the mitochondrial respiratory chain at the level of complex III, induced a substantial increase in ROS formation in this cancer cell line.

In order to characterize the cellular morphological changes induced by the copper(I) compounds, we observed BxPC3 cancer cells treated for 24 h with IC_50_ concentrations of the most representative compound **8** by using TEM. Analogously to what was observed with other Cu(I) complexes [17,20], morphological analysis revealed that **8** induced a massive swelling of the endoplasmic reticulum (ER) membrane, which is a clear sign of ER stress (Figure 4, panel B, c). In addition, at 48 h of treatment, clear mitophagic signs of mitochondria damage and mitophagy induction were evident (Figure 4, panel B, d).

## 3. Materials and Methods

### 3.1. Chemistry

#### 3.1.1. Materials and General Methods

All syntheses and handling were carried out under an atmosphere of dry oxygen-free dinitrogen, using standard Schlenk techniques or a glove box. All solvents were dried, degassed, and distilled prior to use. Elemental analyses (C, H, N, S) were performed with a Fisons Instruments EA-1108 CHNS-O Elemental Analyzer (Thermo Scientific). Melting points were taken on an SMP3 Stuart Scientific Instrument. IR spectra were recorded from 4000 to 400 cm^−1^ on a PerkinElmer Frontier Fourier-transform infrared (FT-IR) instrument, equipped with a single-reflection attenuated total reflection (ATR) unit (universal diamond ATR top-plate) as a sample support. IR annotations used were as follows: br = broad, m = medium, s = strong, sbr = strong broad, sh = shoulder, vbr = very broad, w = weak, wbr = weak broad. ^1^H-, ^13^C-, and ^31^P-NMR spectra were recorded with an Oxford AS400 Varian spectrometer (400.4 MHz for ^1^H, 100.1 MHz for ^13^C, and 162.1 MHz for ^31^P) or with a 500 Bruker Ascend (500.1 MHz for ^1^H, 125 MHz for ^13^C, and 202,4 MHz for ^31^P). Referencing was relative to tetramethylsilane (TMS) (^1^H and ^13^C) and 85% H_3_PO_4_ (^31^P). NMR annotations used were as follows: br = broad; d = doublet, m = multiplet, s = singlet, s br = broad singlet, spt = septept. Electrospray mass spectra (ESI-MS) were obtained in positive- or negative-ion mode on a Series 1100 MSD detector HP spectrometer, using a methanol mobile phase. The compounds were added to reagent grade methanol to give approximately 0.1 mM solutions, injected (1 μL) into the spectrometer via a HPLC HP 1090 Series II fitted with an autosampler, at a flow rate of 300 μL∙min^−1^, employing nitrogen as both drying and nebulizing gas. Capillary voltages were typically 4000 V and 3500 V for the positive- and negative-ion modes, respectively. Confirmation of all major species in this ESI-MS study was aided by comparison of the observed and predicted isotope distribution patterns, with the latter calculated using the IsoPro 3.1 computer program.

All reagents were purchased from Aldrich and used without further purification. The ligands [HC(CO_2_H)(pz)_2_], LH [26], [HC(CO_2_H)(pz^Me2^)_2_], L^2^H [25], L^NMDA^, and L^2NMDA^ [45] were prepared according to literature methods. Their ^13^C-NMR spectra are reported in Figures S2 and S3.

#### 3.1.2. Synthesis of [(LH)Cu(PPh_3_)_2_]PF_6_ (**1**)

Cu(CH_3_CN)_4_PF_6_ (0.373 g, 1.000 mmol) was added to a solution of PPh_3_ (0.525 g, 2.000 mmol) in acetonitrile (50 mL). The reaction mixture was stirred at room temperature for 3 h; then, LH (0.192 g, 1.000 mmol) was added, and the suspension was stirred overnight. The reaction mixture was filtered and dried under reduced pressure; the solid was washed with Et_2_O (50 mL) to remove the excess of PPh_3_ and dried under reduced pressure to give the complex [(LH)Cu(PPh_3_)_2_]PF_6_ (**1**) in 48% yield. Melting point (M.p.) 98–101 °C. ^1^H-NMR (DMSO, 293 K, Figure S4): δ 6.36 (s, 2H, 4-*CH*), 7.26–7.61 (m, 33H, C_6_*H*_5_, C*H*COO, and 5-C*H*), 8.06 (s, 2H, 3-C*H*). ^13^C-NMR (DMSO, 293 K, Figure S4): 74.0 (*C*HCO); 107.2 (4-*C*H); 129.5, 130.8, 132.1, 132.2, 132.7, 133.3, 133.6, 133.9, 134.0 (ArH, 3-*C*H, and 5-*C*H); 141.9 (Ar); 166.2 (CO). ^31^P{H}-NMR (DMSO, 293 K): δ −143.10 (spt, J_F-P_ = 713 Hz), −3.77 (s br). IR (cm^−1^): 3635br (OH); 3138w, 3056w 3004w, 2987w (CH); 1752m (C=O); 1616w, 1586w (C=C/C=N); 1523w, 1481m, 1455w, 1435m, 1403m, 1298m, 1226w, 1207w, 1187w, 1160w, 1096m, 1058w, 1027w, 999w, 986w, 916w, 833s, 741s, 692s. ESI-MS(+) (CH_3_OH), *m/z* (%): 587 (100) [Cu(PPh_3_)_2_]^+^; 517 (60) [(LH)Cu(PPh_3_)]^+^. ESI-MS(−) (CH_3_OH), *m/z* (%): 145 (100) [PF_6_]^−^. Elemental analysis (%): calculated for C_44_H_38_CuF_6_N_4_O_2_P_3_: C, 57.12; H, 4.14; N, 6.06%; found: C, 56.29; H, 4.14; N, 5.58%.

#### 3.1.3. Synthesis of [(L^2^H)Cu(PPh_3_)_2_]PF_6_ (**2**)

Cu(CH_3_CN)_4_PF_6_ (0.373 g, 1.000 mmol) was added to a solution of PPh_3_ (0.525 g, 2.000 mmol) in acetonitrile (50 mL). The reaction mixture was stirred at room temperature for 3 h; then, L^2^H (0.248 g, 1.000 mmol) was added, and the suspension was stirred overnight. The reaction mixture was filtered and dried under reduced pressure; the solid was washed with Et_2_O to remove the excess of PPh_3_ and dried under reduced pressure to give the complex [(L^2^H)Cu(PPh_3_)_2_]PF_6_ (**2**) in 58% yield. M.p. 116–118 °C. ^1^H-NMR (CDCl_3_, 293 K): δ 2.01 (s, 6H, C*H*_3_), 2.47 (s, 6H, C*H*_3_), 6.14 (s, 2H, 4-C*H*), 6.81 (br, 1H, C*H*COO), 7.26–7.68 (m, 30H, C_6_*H*_5_). ^31^P{H}-NMR (CDCl_3_, 293 K): −143.31 (spt, J_F-P_ = 713 Hz), −2.85 (s br). IR (cm^−1^): 3144w, 3053w, 2990w, 2930w (CH); 1740m (C=O); 1586w, 1563m (C=C/C=N); 1482m, 1469sh, 1436w, 1394w, 1314w, 1298sh, 1260w, 1233w, 1181w, 1097m, 1062w, 1047w, 1028w, 999w, 898w, 878w, 832s, 762sh, 744m, 720w, 694s. ESI-MS(+) (CH_3_OH), *m/z* (%): 573 (100) [(L^2^H)Cu(PPh_3_)]^+^. ESI-MS(−) (CH_3_OH), *m/z* (%): 145 (100) [PF_6_]^−^. Elemental analysis (%): calculated for C_48_H_46_CuF_6_N_4_O_2_P_3_: C, 58.75; H, 4.72; N, 5.71%; found: C, 57.58; H, 5.03; N, 5.64%.

#### 3.1.4. Synthesis of [(L^NMDA^)Cu(PPh_3_)_2_]PF_6_ (**3**)

PPh_3_ (0.262 g, 1.000 mmol) was added to a suspension of Cu(CH_3_CN)_4_PF_6_ (0.186 g, 0.500 mmol) in acetonitrile (50 mL). The reaction mixture was stirred at room temperature for 3 h; then, L^NMDA^ (0.222 g, 0.500 mmol) was added, and the suspension was stirred overnight. The reaction mixture was filtered and dried under reduced pressure; then, the solid was dissolved in CH_3_CN and crystallized with a hexane/diethyl ether mixture 2:1. The precipitate was filtered and dried under reduced pressure to give the complex [(L^NMDA^)Cu(PPh_3_)_2_]PF_6_ (**3**) in 88% yield. M.p. 117–120 °C. ^1^H-NMR (CDCl_3_, 293 K, Figure S5): δ 3.33–3.68 (m, 6H, dioxane, and C*H*_2_N), 4.57 (d, 1H, dioxane), 6.35 (m, 2H, 4-C*H*), 7.20–7.68 (m, 45H, C*H*CO, C_6_*H*_5_, 3-C*H*, and 5-C*H*), 8.17 (s br, 1H, N-H). ^31^P{H}-NMR (CDCl_3_, 293 K): −143.75 (spt, J_F-P_ = 714 Hz), 0.25 (s br). IR (cm^−1^): 3639w, 3404w (NH), 3291w; 3122w, 3055w, 2972w, 2936w, 2860w (CH); 2273w, 2098w, 1978w, 1905w, 1823w; 1757w, 1681s (C=O); 1561m, 1519w (C=C/C=N); 1495w, 1481m, 1451m, 1435s, 1402m, 1388m, 1373m, 1350w, 1312m, 1291m, 1271m, 1244m, 1228m, 1210w, 1184w, 1162w, 1138w sh, 1122s, 1096s, 1065m, 1052m, 1026m, 999m, 991m, 959w, 926m, 916m, 834s, 774s, 743s, 693s, 662s. ESI-MS(+) (CH_3_OH), *m/z* (%): 587 (100) [Cu(PPh_3_)_2_]^+^, 768 (20) [(L^NMDA^)Cu(PPh_3_)]^+^, 506 (10) [Cu(L^NMDA^)]^+^, 325 (5) [Cu(PPh_3_)]^+^. ESI-MS(−) (CH_3_OH), *m/z* (%): 145 (100) [PF_6_]^−^. Elemental analysis (%): calculated for C_61_H_55_CuF_6_N_5_O_3_P_3_: C, 62.27; H, 4.71; N, 5.95; found: C, 61.92; H, 4.75; N, 6.49.

#### 3.1.5. Synthesis of [(L^2NMDA^)Cu(PPh_3_)_2_]PF_6_ (**4**)

PPh_3_ (0.157 g, 0.600 mmol) was added to a suspension of Cu(CH_3_CN)_4_PF_6_ (0.112 g, 0.300 mmol) in acetonitrile (50 mL). The reaction mixture was stirred at room temperature for 3 h; then, L^2NMDA^ (0.150 g, 0.300 mmol) was added, and the suspension was stirred overnight. The reaction mixture was filtered and dried under reduced pressure to give the complex [(L^2NMDA^)Cu(PPh_3_)_2_]PF_6_ (**4**) in 70% yield. M.p. 75–78 °C. ^1^H-NMR (CDCl_3_, 293 K): 2.20 (s, 6H, C*H*_3_), 2.60 (s, 6H, C*H*_3_), 3.36–3.75 (m, 6H, dioxane, and C*H*_2_N), 4.61 (d, 1H, dioxane), 6.01 (s br, 1H, 4-C*H*), 6.06 (s br, 1H, 4-C*H*), 6.80 (s, 1H, C*H*CO), 7.25–7.70 (m, 41H, C_6_*H*_5_, N–H). ^31^P{H}-NMR (CDCl_3_, 293 K): −143.99 (spt, J_F-P_ = 714 Hz), −3.85 (s br). IR (cm^−1^): 3394w (NH); 3054w, 2918w, 2853w (CH); 1674m (C=O); 1562m (C=C/C=N); 1478m, 1449sh, 1435m, 1419sh, 1310w, 1270w, 1242w, 1224w, 1182w, 1158w, 1123sh, 1095m, 1060w, 1041w, 1026w, 996w, 983w, 937w, 916w, 835s, 743s, 694s. ESI-MS(+) (CH_3_CN), *m/z* (%): 587 (100) [Cu(PPh_3_)_2_]^+^, 824 (40) [(L^2NMDA^)Cu(PPh_3_)_2_]^+^. ESI-MS(−) (CH_3_CN), *m/z* (%): 145 (100) [PF_6_]^−^. Elemental analysis (%): calculated for C_65_H_63_CuF_6_N_5_O_3_P_3_: C, 63.33; H, 5.15; N, 5.68; found: C, 62.78; H, 4.93; N, 5.42.

#### 3.1.6. Synthesis of [(LH)Cu(PTA)_2_]PF_6_ (**5**)

Cu(CH_3_CN)_4_PF_6_ (0.280 g, 0.750 mmol) was added to a solution (50 mL) of PTA (0.236 g, 1.500 mmol) in acetonitrile. The reaction mixture was stirred at room temperature for 3 h; then, a methanol solution of LH (0.144 g, 0.750 mmol) was added, and the suspension was stirred overnight. The reaction mixture was filtered and dried under reduced pressure to give the complex [(LH)Cu(PTA)_2_]PF_6_ (**5**) in 63% yield. M.p. 109–112 °C. ^1^H-NMR (DMSO, 293 K, Figure S6): δ 4.11 (br, 12H, C*H*_2_P), 4.53–4.67 (m, 12H, NC*H*_2_N), 6.42 (s, 2H, 4-C*H*), 7.29 (s, 1H, C*H*COO), 7.67 (s, 2H, 5-C*H*), 8.04 (s, 2H, 3-C*H*). ^1^H-NMR (D_2_O, 293 K): δ 4.05 (br, 12H, C*H*_2_P), 4.58–4.70 (m, 12H, NC*H*_2_N), 6.48 (s br, 2H, 4-C*H*), 7.15 (s br, 1H, C*H*COO), 7.62 (s, 2H, 5-C*H*), 7.94 (s, 2H, 3-C*H*). ^31^P{H}-NMR (D_2_O, 293 K): δ −145.02 (spt, J_F-P_ = 709 Hz), −87.01 (br). IR (cm^−1^): 3372br (OH); 3124br, 2945br (CH); 1645s (C=O); 1515w (C=C/C=N); 1450w, 1405m, 1359w, 1292s, 1242m, 1207sh, 1119sh, 1100m, 1060w, 1016m, 972m, 950m, 893w, 831s, 748s, 658m. ESI-MS(+) (CH_3_CN), *m/z* (%): 412 (100) [(LH)Cu(PTA)]^+^. ESI-MS(−) (CH_3_CN), *m/z* (%): 145 (100) [PF_6_]^−^; 191 (10) [L]^−^. Elemental analysis (%): calculated for C_20_H_32_CuF_6_N_10_O_2_P_3_: C, 33.60; H, 4.51; N, 19.59; found: C, 32.27; H, 4.38; N, 18.75.

#### 3.1.7. Synthesis of [(L^2^H)Cu(PTA)_2_]PF_6_ (**6**)

Cu(CH_3_CN)_4_PF_6_ (0.280 g, 0.750 mmol) was added to a solution of PTA (0.236 g, 1.500 mmol) in acetonitrile (50 mL). The reaction mixture was stirred at room temperature for 3 h; then, a methanol solution of L^2^H (0.186 g, 0.750 mmol) was added, and the suspension was stirred overnight. The reaction mixture was filtered and dried under reduced pressure; then, the solid was washed with CH_3_CN, and dried under reduced pressure to give the complex [(L^2^H)Cu(PTA)_2_]PF_6_ (**6**) in 44% yield. M.p. 190–193 °C. ^1^H-NMR (DMSO, 293 K, Figure S7): δ 2.19 (s, 6H, C*H*_3_), 2.37 (s, 6H, C*H*_3_), 4.13 (s br, 12H, C*H*_2_P), 4.53–4.82 (m, 12H, NC*H*_2_N), 6.07 (s, 2H, 4-C*H*), 6.44 (s br, 1H, C*H*COO). ^1^H-NMR (D_2_O, 293 K): δ 2.21 (s, 6H, C*H*_3_), 2.40 (s, 6H, C*H*_3_), 4.12 (s br, 12H, C*H*_2_P), 4.63–4.70 (m, 12H, NC*H*_2_N), 6.12 (s, 2H, 4-C*H*), 6.60 (br, 1H, C*H*COO). ^31^P{H}-NMR (D_2_O, 293 K): δ −144.03 (spt, J_F-P_ = 709 Hz), −85.87 (br). ^31^P{H}-NMR (CD_3_CN, 293 K): δ −144.53 (spt, J_F-P_ = 706 Hz), −90.70 (br). IR (cm^−1^): 3384br (OH); 2924br (CH); 1640s (C=O); 1560m (C=C/C=N); 1449w, 1418w, 1393w, 1351w, 1294s, 1242m, 1120w, 1103m, 1043w, 1014m, 971m, 948m, 830s, 741m. ESI-MS(+) (CH_3_CN), *m/z* (%): 468 (100) [(L^2^H)Cu(PTA)]^+^. ESI-MS(−) (CH_3_CN), *m/z* (%): 145 (100) [PF_6_]^−^. Elemental analysis (%): calculated for C_24_H_40_CuF_6_N_10_O_2_P_3_: C, 37.38; H, 5.23; N, 18.16%; found: C, 36.32; H, 4.88; N, 17.01%.

#### 3.1.8. Synthesis of [(L^NMDA^)Cu(PTA)_2_]PF_6_ (**7**)

Cu(CH_3_CN)_4_PF_6_ (0.112 g, 0.300 mmol) was added to a solution of PTA (0.094 g, 0.600 mmol) in acetonitrile (50 mL). The reaction mixture was stirred at room temperature for 3 h; then, a methanol suspension of L^NMDA^ (0.133 g, 0.300 mmol) was added, and the suspension was stirred overnight. The reaction mixture was filtered and dried under reduced pressure to give the complex [(L^NMDA^)Cu(PTA)_2_]PF_6_ (**7**) in 62% yield. M.p. 107–110 °C. ^1^H-NMR (CDCl_3_, 293 K): δ 3.32–3.67 (m, 6H, dioxane, and C*H*_2_N), 4.04 (s br, 12H, C*H*_2_P), 4.38–4.81 (m, 13H, dioxane, and NC*H*_2_N), 6.43 (m, 2H, 4-C*H*), 7.10–8.04 (m, 15H, C_6_*H*_5_, C*H*CO, 3-C*H*, and 5-C*H*), 8.53 (s br, 1H, N*H*). ^1^H-NMR (DMSO, 293 K, Figure S8): δ 3.33–3.80 (m, 6H, dioxane, and C*H*_2_N), 4.06 (s br, 12H, C*H*_2_P), 4.41–4.82 (m, 13H, dioxane, and NC*H*_2_N), 6.45 (m, 2H, 4-C*H*), 7.22–8.05 (m, 15H, C_6_*H*_5_, C*H*CO, 3-C*H*, and 5-C*H*), 8.56 (s br, 1H, N*H*). ^13^C-NMR (DMSO, 293 K, Figure S8): 50.4, 51.8 (*C*H_2_P); 64.0 (*C*HCO); 68.5, 68.7, 71.5, 72.4, 78.8 (dioxane and *C*H_2_N); 107.1 (4-*C*H); 125.6, 127.4, 128.1, 128.4, 128.9 (ArH, 3-*C*H, and 5-*C*H); 142.2, 144.4 (Ar); 164.0 (*C*O). ^31^P{H}-NMR (CD_3_CN, 293 K): δ −143.52 (spt, J_F-P_ = 706 Hz), −91.63 (br). IR (cm^−1^): 3421w (NH); 2925br (CH); 1738sh, 1696br (C=O); 1520br (C=C/C=N); 1449w, 1403w, 1366w, 1292m, 1242m, 1127w, 1100m, 1061w, 1044w, 1015s, 970s, 948s, 917w, 893w, 831s, 757s, 742s, 729m, 700s, 664m. ESI-MS(+) (CN_3_CN) *m/z* (%): 506 (100) [(L^NMDA^)Cu]^+^, 663 (70) [(L^NMDA^)Cu(PTA)]^+^. ESI-MS(−) (CH_3_CN) *m/z* (%): 145 (100) [PF_6_]^−^. Elemental analysis (%): calculated for C_37_H_49_CuF_6_N_11_O_3_P_3_: C, 45.99; H, 5.11; N, 15.94%; found: C, 44.97; H, 4.81; N, 15.07%.

#### 3.1.9. Synthesis of [(L^2NMDA^)Cu(PTA)_2_]PF_6_ (**8**)

Cu(CH_3_CN)_4_PF_6_ (0.112 g, 0.300 mmol) was added to a solution of PTA (0.094 g, 0.600 mmol) in acetonitrile (50 mL). The reaction mixture was stirred at room temperature for 3 h; then, a methanol solution of L^2NMDA^ (0.150 g, 0.300 mmol) was added, and the suspension was stirred overnight. The reaction mixture was filtered and dried under reduced pressure to give the complex [(L^2NMDA^)Cu(PTA)_2_]PF_6_ (**8**) in 59% yield. M.p. 118–121 °C. ^1^H-NMR (CDCl_3_, 293 K): δ 2.06–2.17 (m, 12H, C*H*_3_) 3.24–3.63 (m, 6H, dioxane, and C*H*_2_N), 4.02 (s br, 12H, C*H*_2_P), 4.20–4.62 (m, 13H, dioxane, and NC*H*_2_N), 6.19 (s, 1H, 4-C*H*), 6.21 (s, 1H, 4-C*H*), 6.98 (s, 1H, C*H*CO), 7.22–7.46 (m, 10H, C_6_*H*_5_), 9.01 (s br, 1H, N*H*). ^1^H-NMR (DMSO, 293 K, Figure S9): δ 2.03–2.26 (m, 12H, C*H*_3_) 3.32–3.78 (m, 6H, dioxane, and C*H*_2_N), 4.04 (s br, 12H, C*H*_2_P), 4.23–4.65 (m, 12H, NC*H*_2_N), 4.81(d, 1H, dioxane), 6.19 (s, 1H, 4-C*H*), 6.23 (s, 1H, 4-C*H*), 7.08 (s br, 1H, C*H*CO), 7.23–7.49 (m, 10H, C_6_*H*_5_), 9.25 (s br, 1H, N*H*). ^13^C-NMR (DMSO, 293 K, Figure S9): 14.2 (*C*H_3_); 14.4 (*C*H_3_); 50.7, 51.9 (*C*H_2_P); 64.0 (*C*HCO); 68.7, 71.5, 72.4, 78.8 (dioxane and *C*H_2_N); 107.1 (4-*C*H); 125.6, 127.5, 128.0, 128.4, 128.9 (ArH); 142.2, 142.8, 144.3, 150.6 (Ar, 3-*C*, and 5-*C*); 164.2 (*C*O). ^31^P{H}-NMR (CD_3_CN, 293 K): δ −143.52 (spt, J_F-P_ = 706 Hz), −89.0 (br). IR (cm^−1^): 3420w (NH); 2921br (CH); 1699br (C=O); 1563w (C=C/C=N); 1519br, 1449m, 1416m, 1315w, 1293m, 1242s, 1127w, 1102m, 1062w, 1045w, 1014s, 959s, 947s, 893w, 873w, 830s, 800s, 741s, 731s, 700s, 664m. ESI-MS(+) (CH_3_CN), *m/z* (%): 719 (100) [(L^2NMDA^)Cu(PTA)]^+^. ESI-MS(−) (CH_3_CN) *m/z* (%): 145 (100) [PF_6_]^−^. Elemental analysis (%): calculated for C_41_H_57_CuF_6_N_11_O_3_P_3_: C, 48.16; H, 5.62; N, 15.07; found: C, 47.46; H, 5.55; N, 14.32. 

### 3.2. Experiments with Cultured Human Cells

Copper complexes **1–8** and relative free ligands were dissolved in DMSO just before the experiment, and a calculated amount of drug solution was added to the cell growth medium to a final solvent concentration of 0.5%, which had no detectable effects on cell viability.

Cisplatin and doxorubicin were dissolved in 0.9% sodium chloride solution. MTT (3-(4,5-dimethylthiazol-2-yl)-2,5-diphenyltetrazolium bromide), doxorubicin, and cisplatin were purchased from Sigma Chemical Co, St. Louis, MO, USA.

#### 3.2.1. Cell Cultures

Human lung (H157), breast (MCF-7), and pancreatic (BxPC3 and PSN-1) carcinoma cell lines were obtained from the American Type Culture Collection (ATCC, RocKville, MD). A431 and A431-Pt are cisplatin-sensitive and -resistant human cervical carcinoma cells that were kindly provided by Prof. F. Zunino (Division of Experimental Oncology B, Istituto Nazionale dei Tumori, Milan, Italy). The A2780 cells and multi-drug-resistant variant, A2780 ADR, are human ovarian adenocarcinoma cell lines that were kindly provided by Prof. A. Colabufo (Dipartimento di Farmacia, Università di Bari, Italy). A2780cis cells are human ovarian adenocarcinoma cells resistant to cisplatin that were kindly provided by Prof. M.P. Rigobello (Department of Biomedical Sciences, University of Padova). Cell lines were maintained in the logarithmic phase at 37 °C in a 5% carbon dioxide atmosphere using Roswell Park Memorial Institute (RPMI)-1640 cell culture medium containing 10% fetal calf serum (Euroclone, Milan, Italy), antibiotics (50 units/mL penicillin and 50 μg/mL streptomycin), and 2 mM l-glutamine.

#### 3.2.2. MTT Assay

The growth inhibitory effect toward 2D tumor cell lines was evaluated by means of the MTT assay as previously described [49]. IC_50_ values were calculated using a four-parameter logistic (4-PL) model. MTT data are the mean of no fewer than three (3–5) independent experiments, and each independent experiment was performed in triplicate.

#### 3.2.3. Spheroid Cultures

Spheroid cultures were obtained by seeding 2.5 × 10^3^ BxPC3 or H157 cancer cells/well in round-bottom non-tissue culture-treated 96-well plate (Greiner Bio-one, Kremsmünster, Austria) in phenol red-free RPMI-1640 medium (Sigma Chemical Co.), contaiNning 10% FCS and supplemented with 20% methyl cellulose stock solution.

#### 3.2.4. APH Assay

An APH modified assay was used for determining cell viability in 3D spheroids, as previously described [12]. IC_50_ values were calculated using a four-parameter logistic (4-PL) model.

#### 3.2.5. Comet Assay

About 4 × 10^4^ BxPC3 cells were seeded in 25-cm^2^ flasks in growth medium (6 mL). After 24 h, cells were incubated for 3h with 2.5 µM of tested compounds. Cells were washed twice with cold PBS, harvested, and centrifuged; then, DNA fragmentation was measured using the alkaline comet assay. Low-melting-point agarose, 300 μL (Trevigen Inc., Gaithersburg, MD, USA), was heated to 37 °C and combined with 2 × 10^5^ cells per mL of cell suspension. Each well of a 20-well CometSlide was filled with 30 μL of the cell/agarose suspension. The slides were placed in a 4 °C refrigerator in the dark for 15 min to solidify. Slides were then immersed in 50 mL of pre-chilled lysis solution containing Trizma base, Triton X-100, and DMSO; then, they were left at 4 °C for 30 min to facilitate cell membrane and histone removal. After draining excess liquid, the slides were transferred to 50 mL of freshly prepared (same day) alkaline DNA unwinding solution (200 mmol/L NaOH, 1 mmol/L ethylenediaminetetraacetic acid (EDTA), pH > 13) and incubated at room temperature in the dark for 20 min. After the unwinding step, electrophoresis was performed at 21 V for 30 min. Slides were then rinsed with distilled water and fixed 5 min in 70% ethanol. Slides were dried and stained for 5 min at 4  °C with SYBR Green I (Trevigen, Inc., Gaithersburg, MD, USA), diluted 1:10 000 in 10 mmol/L Tris pH 7.5, 1 mmol/L EDTA; then, they were drained to remove excess staining solution and thoroughly dried at room temperature in the dark. Micrographs were taken with a Zeiss LSM-800 confocal microscope (Zeiss, Oberkochen, Germany). All photos were typeset in Zen 2.3 system (Zeiss, Oberkochen, Germany).

#### 3.2.6. ROS Production

The production of ROS was measured in BxPC3 cells (10^4^ per well) grown for 24 h in a 96-well plate in RPMI medium without phenol red (Sigma Chemical Co.). Cells were then washed with PBS and loaded with 10 μM 5-(and-6)-chloromethyl-2′,7′-dichlorodihydrofluorescein diacetate acetyl ester (CM-H_2_DCFDA) (Molecular Probes-Invitrogen, Eugene, OR) for 25 min, in the dark. Afterward, cells were washed with PBS and incubated with IC_50_ concentrations of tested compounds. Fluorescence increase was estimated utilizing the wavelengths of 485 nm (excitation) and 527 nm (emission) in a Fluoroskan Ascent FL (Labsystem, Vantaa, Finland) plate reader. Antimycin (3 μM, Sigma Chemical Co), a potent inhibitor of Complex III in the electron transport chain, and auranofin were used as positive controls.

#### 3.2.7. TEM Analyses

About 10^6^ BxPC3 cells were seeded in 24-well plates and, after 24 h of incubation, they were treated with the tested compounds and incubated for additional 24 h. Cells were then washed with cold PBS, harvested, and directly fixed with 1.5% glutaraldehyde buffer with 0.2 M sodium cacodylate, pH 7.4. After washing with buffer and post-fixation with 1% OsO_4_ in 0.2 M cacodylate buffer, specimens were dehydrated and embedded in epoxy resin (Epon Araldite, Electron Microscopy Sciences, Fort Washington, PA, USA). Sagittal serial sections (1 μm) were counterstained with toluidine blue; thin sections (90 nm) were given contrast by staining with uranyl acetate and lead citrate. Micrographs were taken with a Hitachi H-600 electron microscope (Hitachi, Tokyo, Japan) operating at 75 kV. All photos were typeset in Corel Draw 11.

#### 3.2.8. Cellular Uptake

BxPC3cells (3 × 10^6^) were seeded in 75-cm^2^ flasks in growth medium (20 mL). After overnight incubation, the medium was replaced, and the cells were treated with tested compounds for 24 h. Cell monolayers were then processed and mineralized as previously described [29]. The sample was analyzed for copper by using a Varian AA Duo graphite furnace atomic absorption spectrometer (Varian, Palo Alto, CA; USA) at the wavelength of 324 nm. The calibration curve was obtained using known concentrations of standard solutions purchased from Sigma Chemical Co. purchased from Sigma Chemical Co.

#### 3.2.9. Statistical Analysis

All values are the means ± SD of no fewer than three measurements starting from three different cell cultures. Multiple comparisons were made by ANOVA followed by the Tukey–Kramer multiple comparison test (* *p* < 0.05, ** *p* < 0.01), using GraphPad software.

## 4. Conclusions

In the present study, new Cu(I) complexes **1–8** were prepared. The biofunctionalizable ligands LH and L^2^H were selected as coordinating agents, due to the κ^3^-NNO coordination behavior of bis(azol-1-yl) methanes and to the presence of a carboxylic function suitable for the coupling with bioactive molecules. The derivatives L^NMDA^ and L^2NMDA^, obtained via the conjugation of LH and L^2^H with a potent NMDA receptor antagonist, were also used as coordinating agents. Phosphane ligands endowed with different lipophilicity, such as PPh_3_ and PTA, were selected as coligands to stabilize copper in the +1 oxidation state.

All the investigated complexes showed a significant cytotoxic effect in a panel of human tumor cell lines, with IC_50_ values in the low- and sub-micromolar range, which were in general significantly lower than that of the uncoordinated ligands and the reference compound cisplatin. Moreover, they were able to overcome the cisplatin and multi-drug resistance.

Interestingly, all the complexes showed very high cytotoxic activity even in 3D spheroids of H157 and BxPC3 cancer cells, which more closely mimic the heterogeneity and complexity of in vivo tumors, being consequently more predictive for in vivo results than conventional 2D cell cultures.

All the compounds were also able to cross cellular plasmalemma, and copper accumulated in treated cancer cells, with a partially direct and linear correlation between the cytotoxic activity and the cellular uptake.

Cellular DNA studies revealed that the complexes were only slightly effective in inducing cleavage of nuclear DNA. Moreover, no tested complexes induced a significant increase in basal ROS production, excluding the hypothesis that they induce direct or indirect DNA damage via an oxidative route.

Finally, morphological analysis highlighted that complex **8**, the most active against 3D spheroids of BxPC3 cells, induced a massive swelling of the ER membrane, which is a clear sign of ER stress.

Due to their interesting biological profile, these new complexes may be considered promising lead compounds for the development of novel antitumor agents as potential alternatives to cisplatin and, in general, to platinum-based drugs.

## Figures and Tables

**Table 1:** In vitro antitumor activity of **1–8** and relative ligands, cisplatin, and doxorubicin ^a^.

	IC_50_ ± SD (μM)											
	PSN-1	BxPC3	MCF-7	H157	A431	A431-Pt	RF ^b^	A2780	A2780cis	RF ^b^	A2780 ADR	RF ^b^
**1**	3.1 ± 1.9	0.3 ± 0.05	1.0 ± 0.1	1.3 ± 0.3	0.6 ± 0.1	1.1 ± 0.4	1.7	0.5 ± 0.3	0.7 ± 0.1	1.6	0.5 ± 0.1	1.2
**2**	1.6 ± 0.2	0.1 ± 0.05	1.8 ± 0.01	1.1 ± 0.2	2.7 ± 0.5	1.3 ± 0.1	0.5	0.6 ± 0.17	0.7 ± 0.2	1.0	0.9 ± 0.3	1.5
**3**	0.4 ± 0.04	0.02 ± 0.01	0.8 ± 0.1	0.4 ± 0.1	0.2 ± 0.01	0.1 ± 0.01	0.7	0.1 ± 0.01	0.1 ± 0.01	0.5	0.2 ± 0.02	1.7
**4**	1.7 ± 0.7	0.07 ± 0.02	2.5 ± 0.6	1.1 ± 0.5	0.6 ± 0.1	1.0 ± 0.2	1.7	0.2 ± 0.1	0.19 ± 0.05	1.3	0.3 ± 0.1	1.9
**5**	1.2 ± 0.04	0.3 ± 0.01	1.0 ± 0.1	1.0 ± 0.1	2.8 ± 1.2	1.3 ± 0.2	0.5	0.2 ± 0.05	0.3 ± 0.2	1.5	0.2 ± 0.1	1.2
**6**	0.02 ± 0.01	0.04 ± 0.02	0.6 ± 0.03	2.3 ± 0.5	0.2 ± 0.03	0.3 ± 0.6	1.7	0.9 ± 0.2	1.1 ± 0.3	1.2	1.3 ± 0.2	1.5
**7**	1.0 ± 0.3	0.2 ± 0.02	0.5 ± 0.03	0.4 ± 0.2	3.3 ± 0.7	5.9 ± 1.5	1.8	0.4 ± 0.04	0.4 ± 0.1	1.1	0.4 ± 0.1	1.1
**8**	0.6 ± 0.3	0.3 ± 0.06	1.8 ± 0.1	0.8 ± 0.2	3.2 ± 0.3	1.9 ± 0.5	0.6	0.04 ± 0.01	0.01 ± 0.01	0.3	0.1 ± 0.02	1.3
**LH**	>50	21.2 ± 3.2	>50	19.4 ± 2.6	>50	-	-	>50	-		-	-
**L^2^H**	>50	>50	>50	21.4 ± 3.0	>50	-	-	45.5 ± 7.3	-		-	-
**NMDA**-**ANT**	10.4 ± 2.6	8.3 ± 2.7	12.1 ± 3.2	5.3 ± 0.7	11.2 ± 2.3	-	-	19.8 ± 3.3	-		-	-
**L^NMDA^**	9.6 ± 2.3	13.1 ± 3.5	7.4 ± 2.2	6.6 ± 1.4	7.6 ± 0.9	-	-	15.5 ± 2.6	-		-	-
**L^2NMDA^**	>50	42.1 ± 3.0	>50	>50	>50	-	-	>50	-		-	-
**Cisplatin**	12.1 ± 2.9	7.3 ± 1.2	8.8 ± 0.2	26.7 ± 3.2	1.4 ± 0.3	2.9 ± 0.6	2.0	0.45 ± 0.1	2.6 ± 0.2	5.8	-	-
**Doxorubicin**	-	-	-	-	-	-	-	0.004 ± 0.001	-		0.09 ± 0.01	21.5

^a^ Cells (3–8 × 10^3^ mL^−1^) were treated for 72 h with increasing concentrations of tested compounds. Cytotoxicity was assessed by 3-(4,5-dimethylthiazol-2-yl)-2,5-diphenyltetrazolium bromide (MTT) test. Half maximal inhibitory concentration (IC_50_) values were calculated using a four-parameter logistic model (*p* < 0.05). SD = standard deviation. ^b^ RF = IC_50_ resistant/IC_50_ parental cell lines.

**Table 2:** Activity of **1–8** and cisplatin in three-dimensional (3D) cell cultures ^a^.

	IC_50_ ± SD (μM)	
	H157	BxPC3
**1**	4.7 ± 0.3	3.1 ± 0.5
**2**	3.3 ± 1.0	2.4 ± 0.2
**3**	10.9 ± 3.0	4.5 ± 1.3
**4**	2.7 ± 0.5	6.5 ± 1.1
**5**	11.9 ± 2.0	10.2 ± 2.3
**6**	10.0 ± 0.4	5.5 ± 0.9
**7**	13.0 ± 1.1	8.5 ± 0.9
**8**	2.9 ± 0.5	1.2 ± 0.2
**Cisplatin**	52.51 ± 1.31	100.5 ± 12.1

^a^ Spheroids (2.5 × 10^3^ cells/well) were treated for 72 h with increasing concentrations of tested compounds. The growth-inhibitory effect was evaluated by means of the acid phosphatase (APH) test. IC_50_ values were calculated from the dose–survival curves using a four-parameter logistic model (*p* < 0.05). SD = standard deviation.

## Supplementary Materials

Supplementary materials can be found at https://www.mdpi.com/1422-0067/21/7/2616/s1. Figure S1: Spectra of solutions containing ctDNA (0.14 mM) and increasing concentrations of ligands and related complexes **1–8** in Tris-HCl buffer, pH = 7.3; Figure S2: ^13^C-NMR spectra of L^H^ and L^2^H; Figure S3: ^13^C-NMR spectra of L^NMDA^ and L^2NMDA^; Figure S4: ^1^H-NMR and ^13^C-NMR spectra of **1**; Figure S5: ^1^H-NMR spectrum of **3**; Figure S6: ^1^H-NMR spectrum of **5**; Figure S7: ^1^H-NMR spectrum of **6**; Figure S8: ^1^H-NMR, ^13^C-NMR, and C/H COSY spectra of **7**; Figure S9: ^1^H-NMR, ^13^C-NMR, and C/H COSY spectra of **8**.

## Abbreviations

PPh_3_	triphenylphosphine
PTA	1,3,5-triaza-7-phosphaadamantane
MDR	multi-drug-resistant
TEM	transmission electron microscopy
ROS	reactive oxygen species
CDI	carbonyldiimidazole
APH	acid phosphatase
ER	endoplasmic reticulum
