# Supplementary material for: Synthesis and Cytotoxic Activity Evaluation of New Cu(I) Complexes of Bis(pyrazol-1-yl) Acetate Ligands Functionalized with an NMDA Receptor Antagonist
Source: Int J Mol Sci. 2020 Apr 9;21(7):2616. doi: 10.3390/ijms21072616 (PMC7178194; doi:10.3390/ijms21072616)
Supplement: Supplementary file 1 [file ijms-21-02616-s001.pdf]

(Supplementary material)

Synthesis and cytotoxic activity evaluation of new Cu(I) complexes of bis(pyrazol-1-yl)acetate ligands functionalized with an NMDA receptor antagonist

Maura Pellei, Luca Bagnarelli, Lorenzo Luciani, Fabio Del Bello,* Gianfabio Giorgioni, Alessandro Piergentili, Wilma Quaglia, Michele De Franco, Valentina Gandin,* Cristina Marzano, Carlo Santini

Table of Contents:

-**Figure S1**: Spectra of solutions containing CT-DNA (0.14 mM) and increasing concentrations of ligands and related complexes **1-8** in Tris-HCl buffer, pH =7.3.

-**Figure S2**: ^{13}C NMR spectra of L^H and L^2H.

-**Figure S3**: ^{13}C NMR spectra of L^{NMDA} and L^{2NMDA}.

-**Figure S4**: ^{1}H NMR and ^{13}C NMR spectra of **1**.

-**Figure S5**: ^{1}H NMR spectrum of **3**.

-**Figure S6**: ^{1}H NMR spectrum of **5**.

-**Figure S7**: ^{1}H NMR spectrum of **6**.

-**Figure S8**: ^{1}H NMR, ^{13}C NMR and C/H COSY spectra of **7**.

-**Figure S9**: ^{1}H NMR, ^{13}C NMR and C/H COSY spectra of **8**.

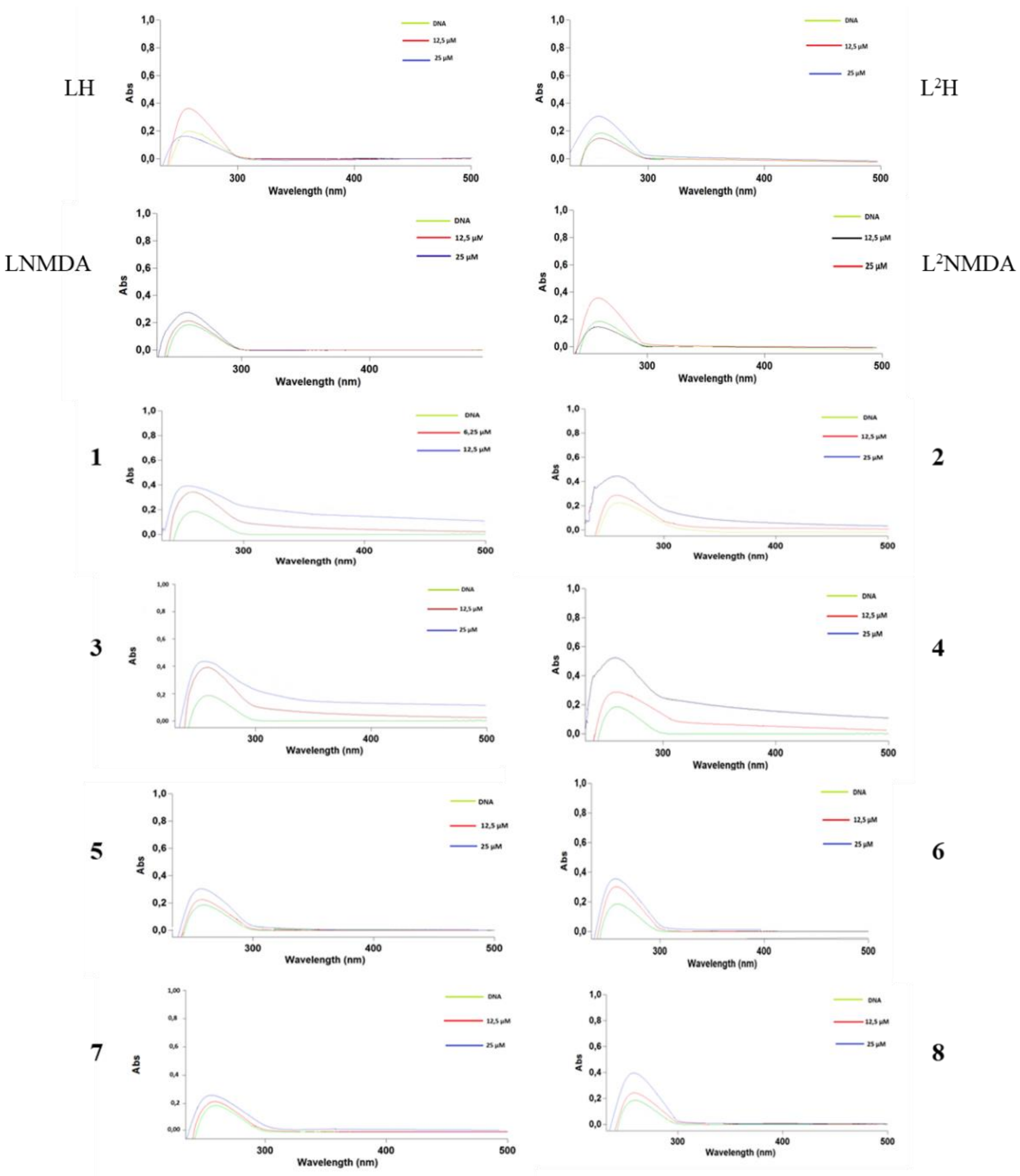

Figure S1. Spectra of solutions containing CT-DNA (0.14 mM) and increasing concentrations of ligands and related complexes **1-8** in Tris-HCl buffer, pH =7.3.

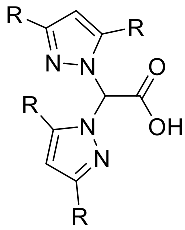

LH: R = H
L^2H: R = Me

A

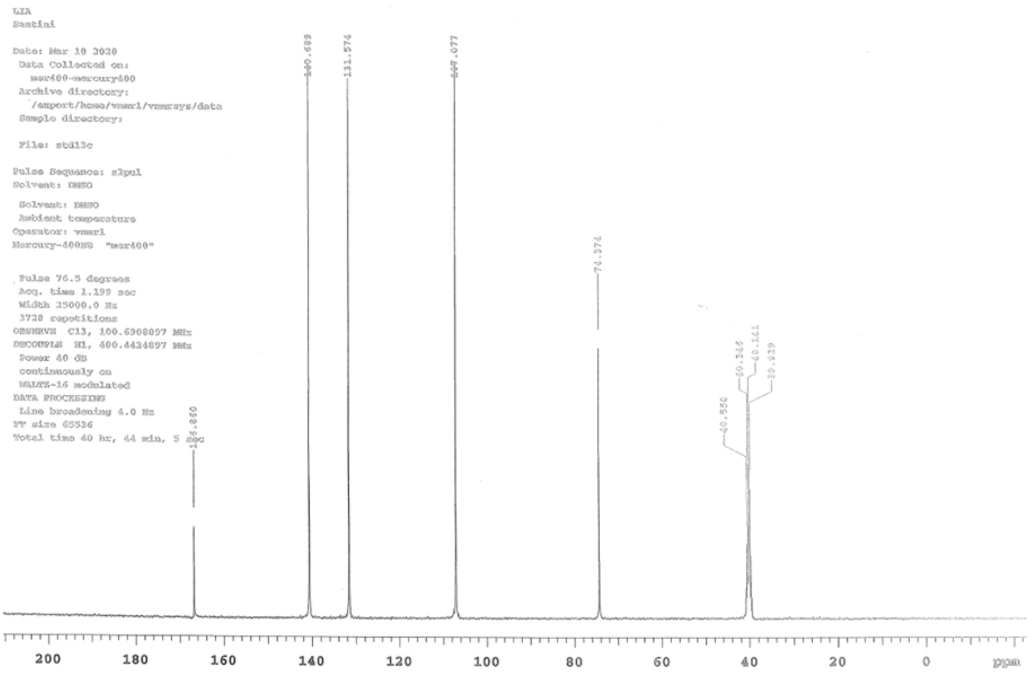

B

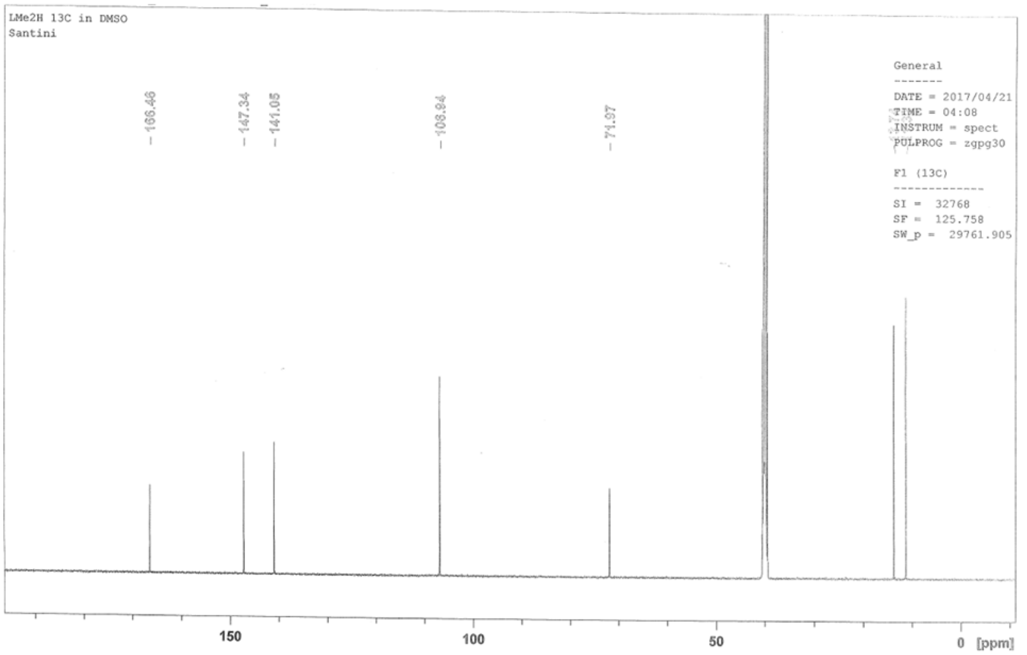

Figure S2. ^{13}C NMR spectra of (A) LH and (B) L^2H.

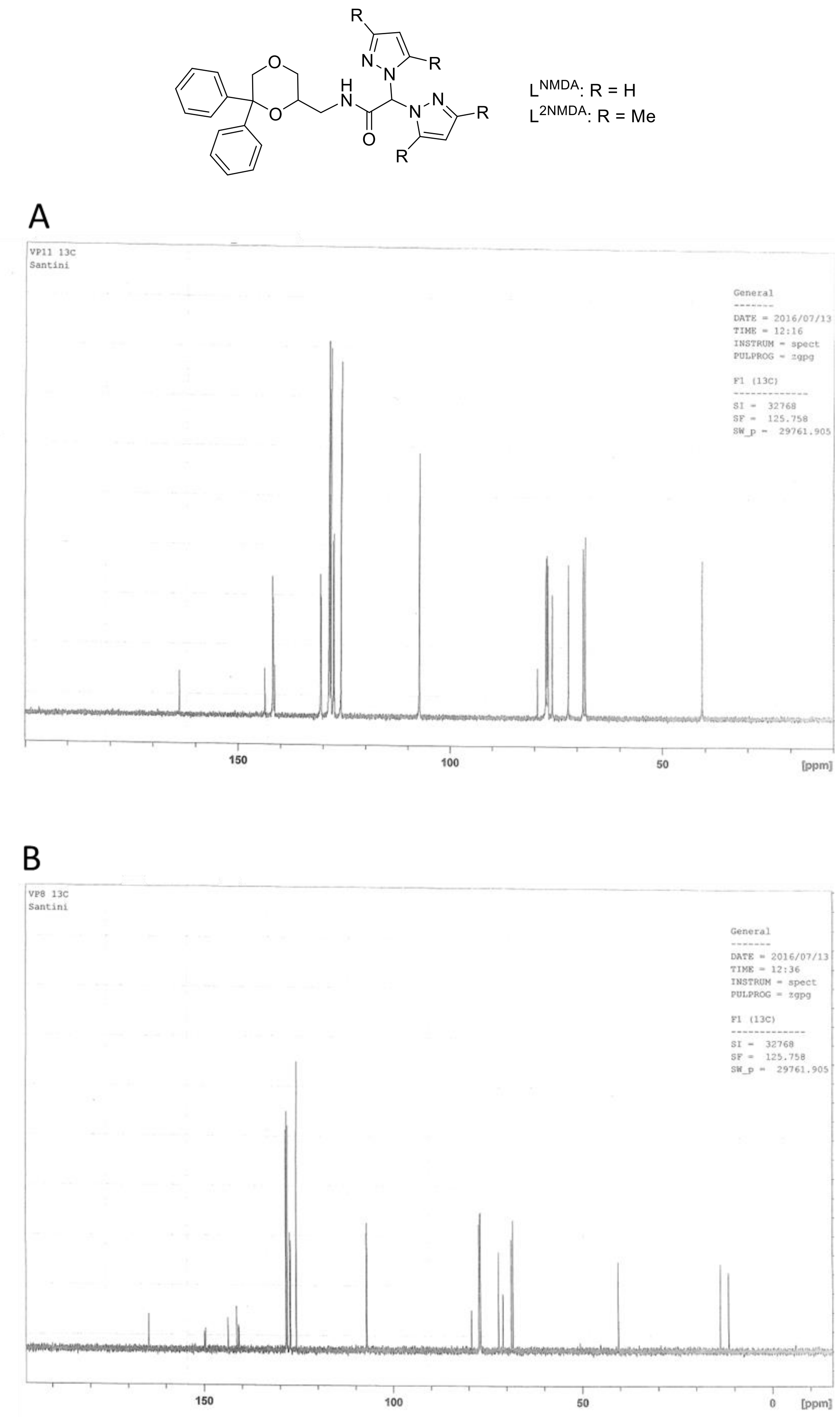

Figure S3: ^{13}C NMR spectra of (A) L^{NMDA} and (B) L^{2NMDA}.

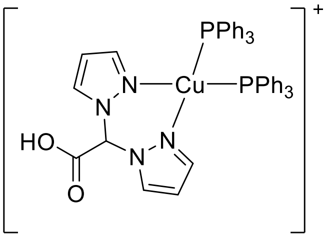

1

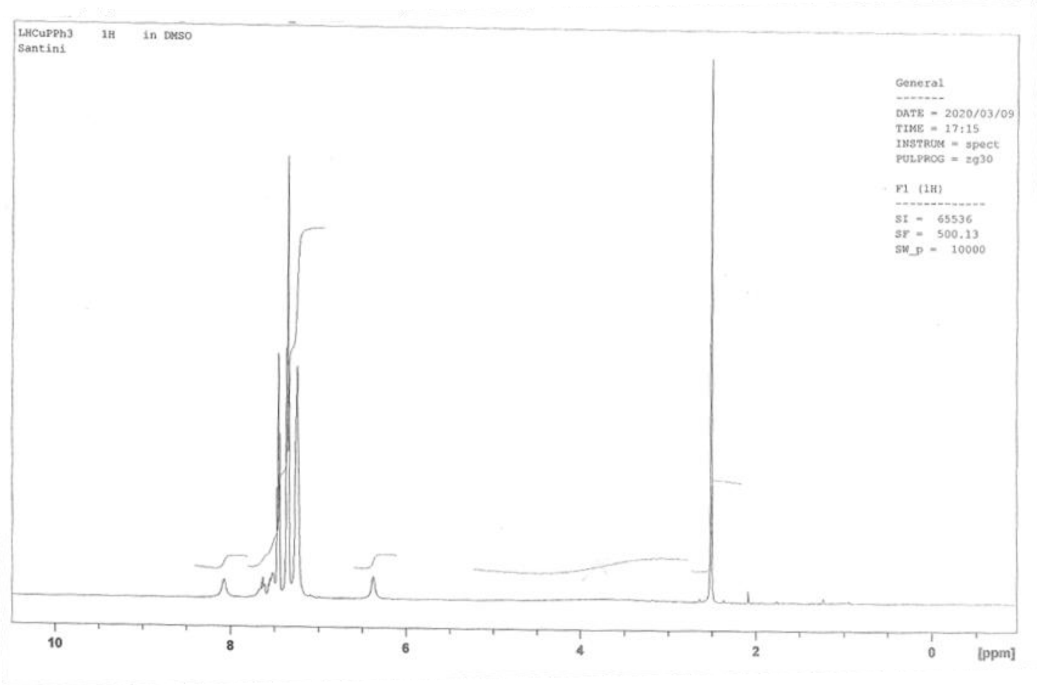

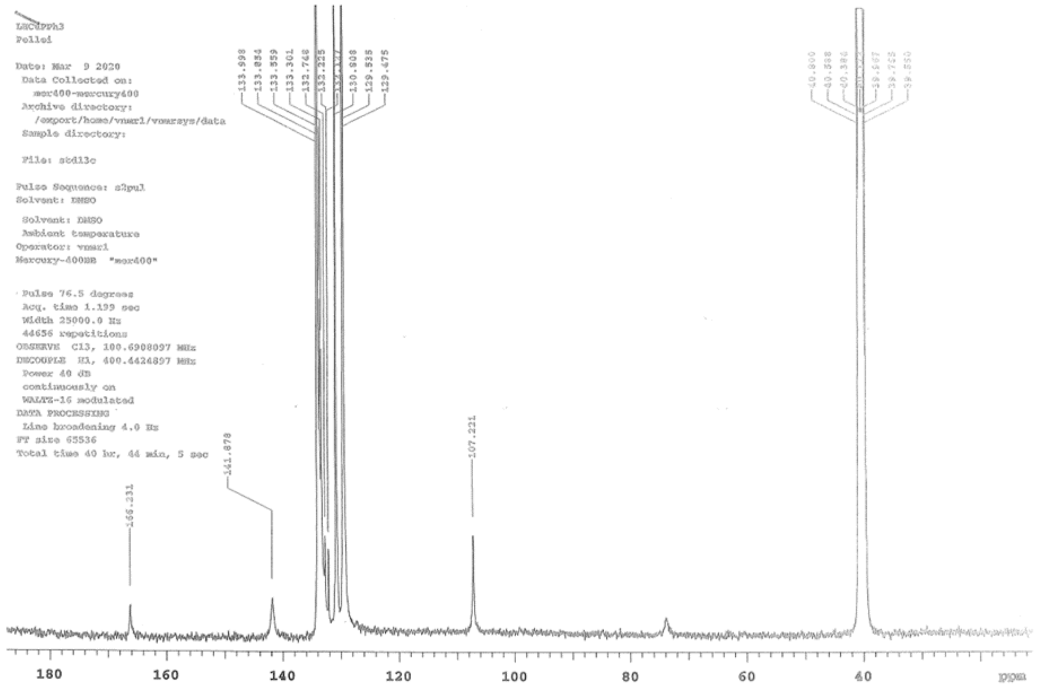

Figure S4: ^{1}H NMR and ^{13}C NMR spectra of **1**

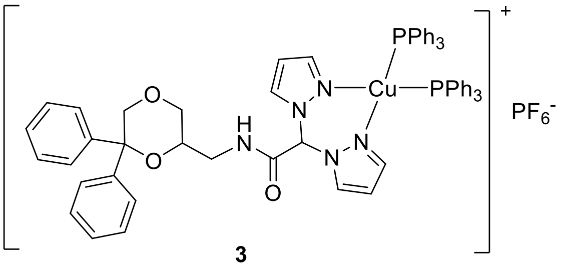

3

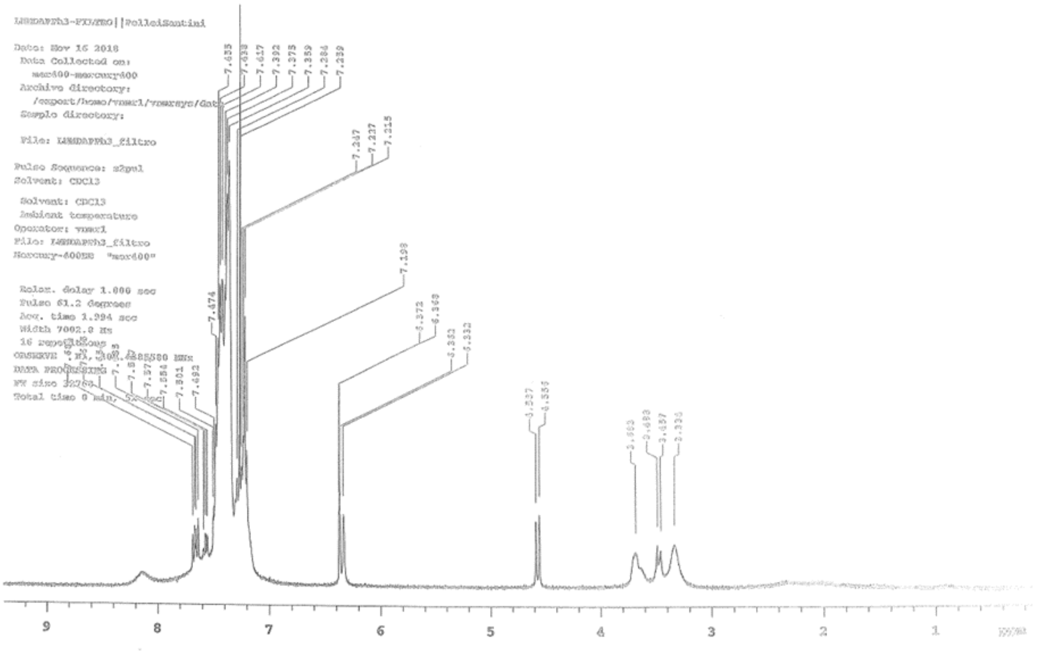

Figure S5: ^{1}H NMR spectrum of **3**

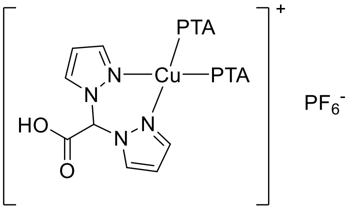

5

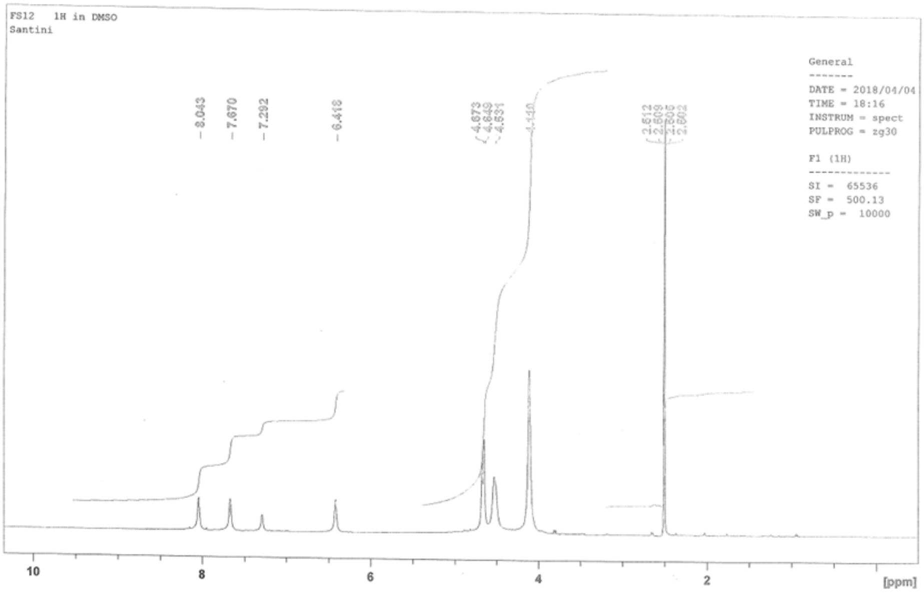

Figure S6: ^{1}H NMR spectrum of **5**

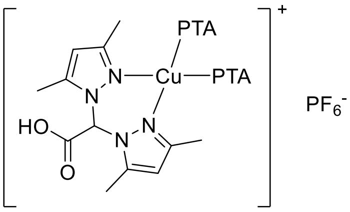

6

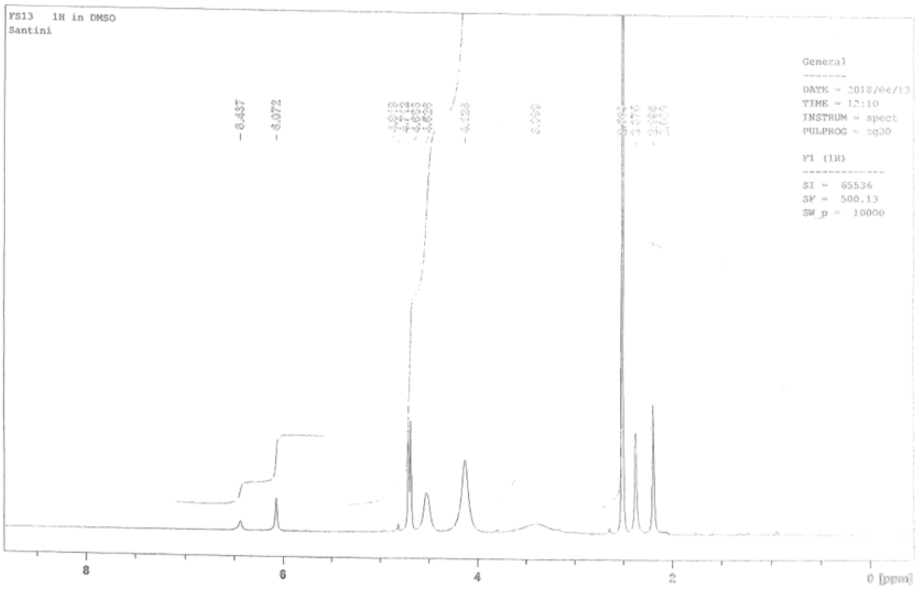

Figure S7: ^{1}H NMR spectrum of **6**

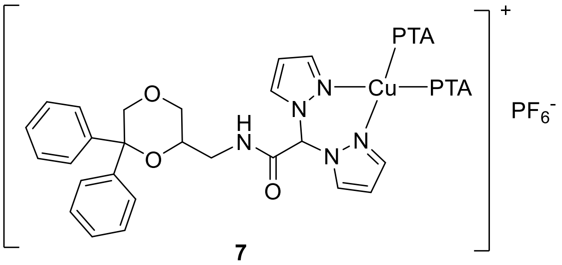

PTA
Cu
PTA
PF6-

7

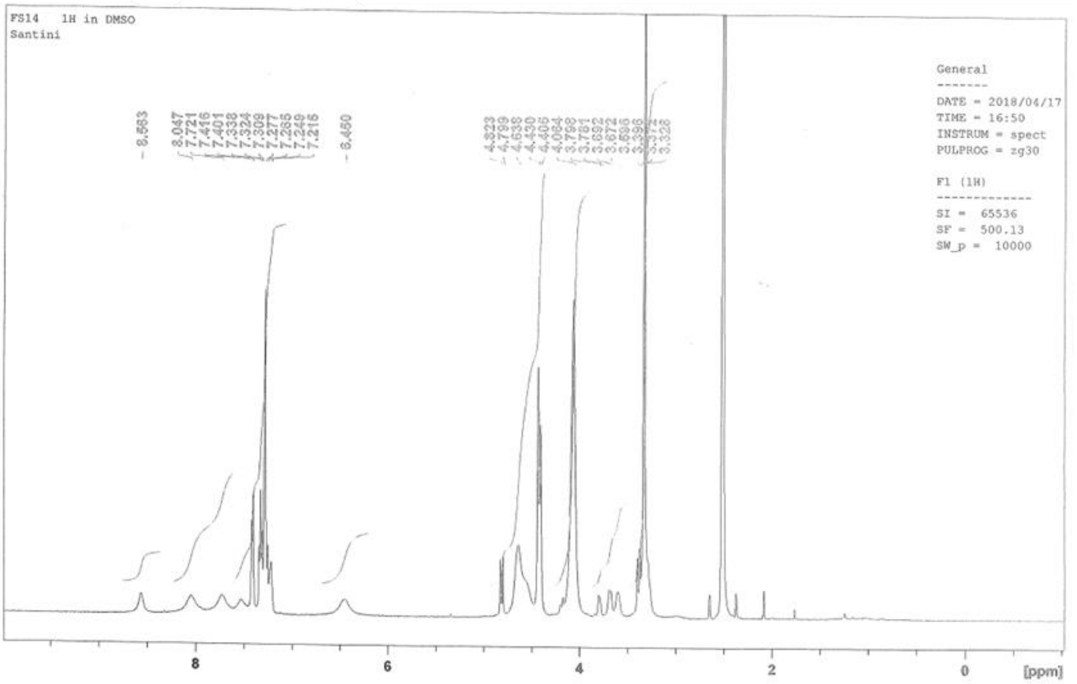

FS14 1H in DMSO
Santini
General
DATE = 2018/04/17
TIME = 16:50
INSTRUM = spect
PULPROG = zg30
F1 (1H)
SI = 65536
SF = 500.13
SW_p = 10000
8
6
4
2
0
[ppm]

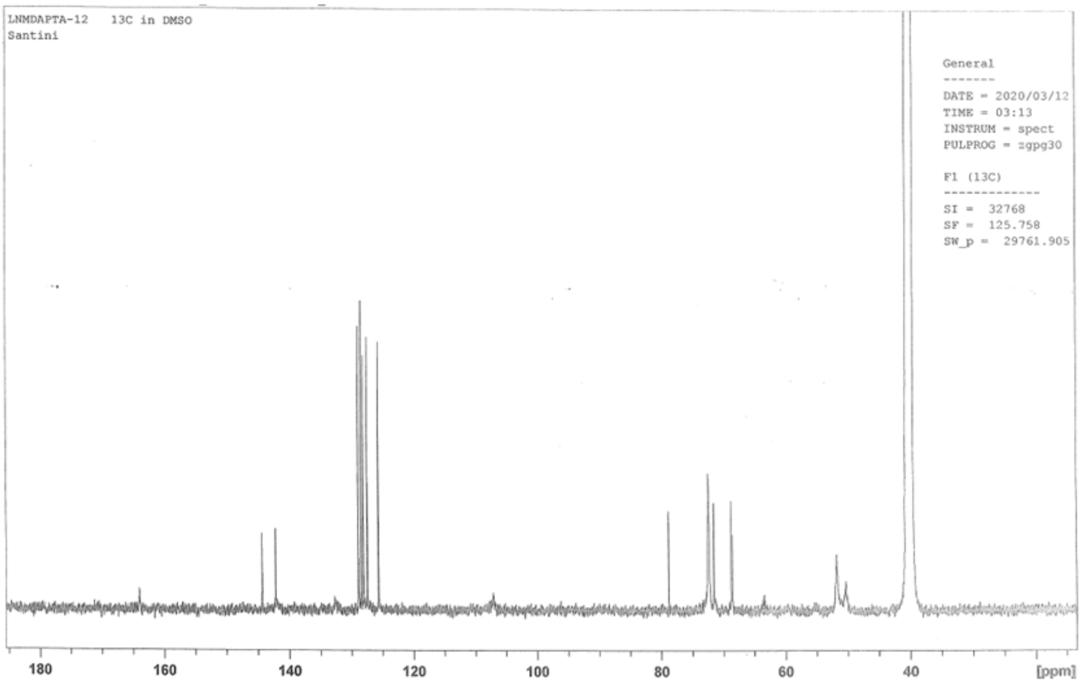

LNMDAPTA-12 13C in DMSO
Santini
General
DATE = 2020/03/12
TIME = 03:13
INSTRUM = spect
PULPROG = zgpg30
F1 (13C)
SI = 32768
SF = 125.758
SW_p = 29761.905
180
160
140
120
100
80
60
40
[ppm]

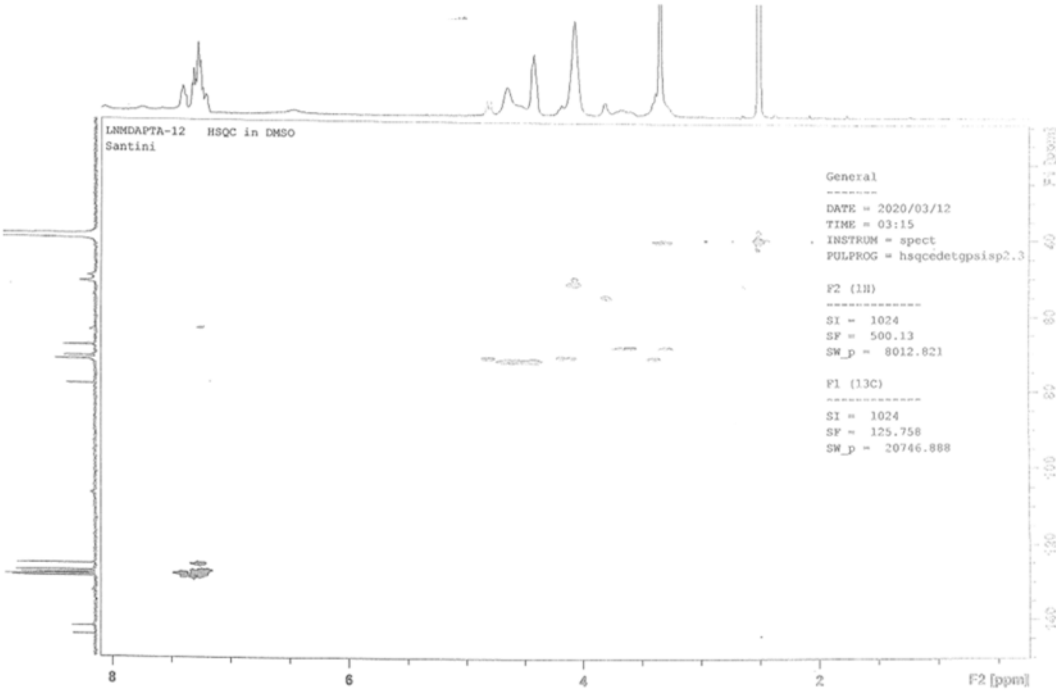

Figure S8: ^{1}H NMR, ^{13}C NMR and C/H COSY spectra of **7**

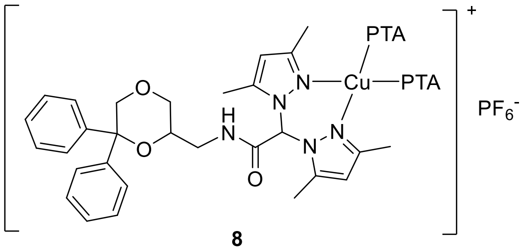

PTA
PTA
Cu
PF_6^-

8

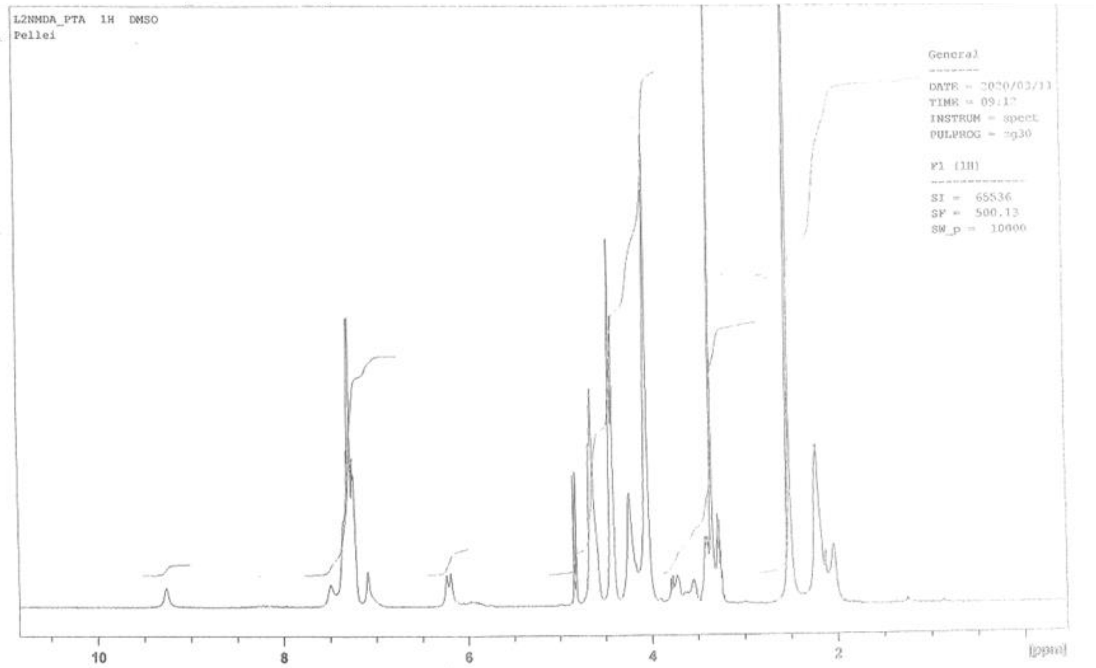

L2NMDA_PTA 1H DMSO
Pellei
General
DATE = 2020/03/11
TIME = 09:12
INSTRUM = spect
PULPROG = zg30
F1 (1H)
SI = 65536
SF = 500.13
SW_p = 10000
10
8
6
4
2
[ppm]

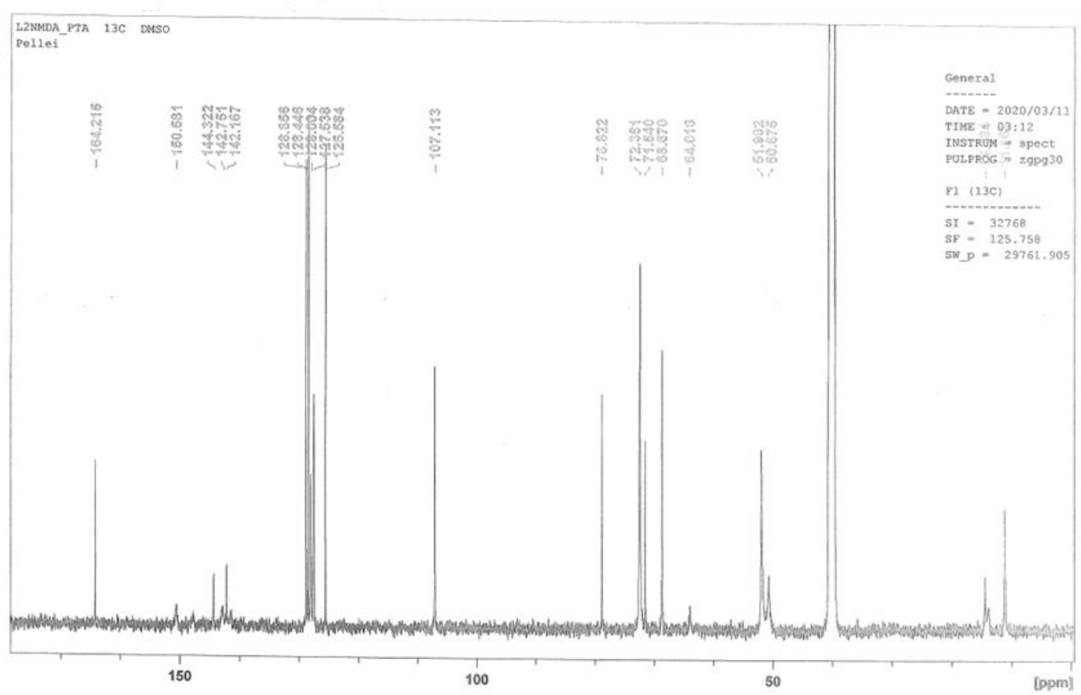

L2NMDA_PTA 13C DMSO
Pellei
164.216
150.581
144.322
142.751
142.167
128.558
128.448
128.004
127.638
125.584
107.113
78.822
72.381
71.640
68.870
64.016
51.902
50.676
General
DATE = 2020/03/11
TIME = 03:12
INSTRUM = spect
PULPROG = zgpg30
F1 (13C)
SI = 32768
SF = 125.758
SW_p = 29761.905
150
100
50
[ppm]

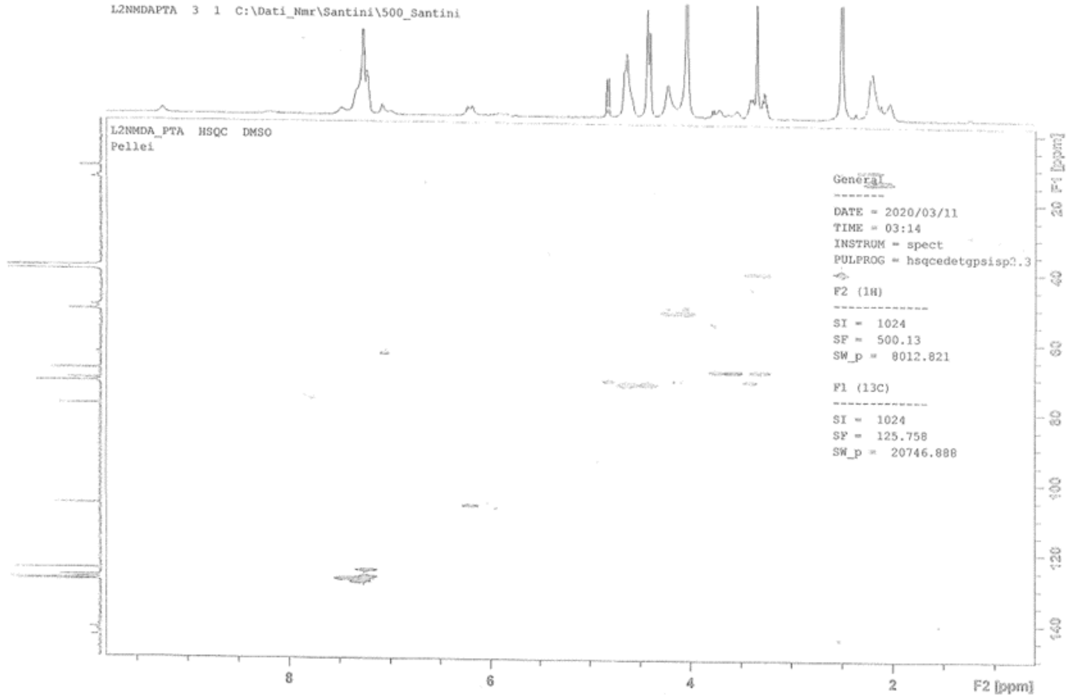

Figure S9: ^{1}H NMR, ^{13}C NMR and C/H COSY spectra of **8**
